# Deep Learning for Diabetic Retinopathy Analysis: A Review, Research Challenges, and Future Directions

**DOI:** 10.3390/s22186780

**Published:** 2022-09-08

**Authors:** Muhammad Waqas Nadeem, Hock Guan Goh, Muzammil Hussain, Soung-Yue Liew, Ivan Andonovic, Muhammad Adnan Khan

**Affiliations:** 1 Faculty of Information and Communication Technology (FICT), Universiti Tunku Abdul Rahman (UTAR), Kampar 31900, Malaysia; 2 Department of Computer Science, School of Systems and Technology, University of Management and Technology, Lahore 54000, Pakistan; 3 Department of Electronic and Electrical Engineering, Royal College Building, University of Strathclyde, 204 George St., Glasgow G1 1XW, UK; 4 Pattern Recognition and Machine Learning Lab, Department of Software, Gachon University, Seongnam 13557, Korea; 5 Faculty of Computing, Riphah School of Computing and Innovation, Riphah International University, Lahore Campus, Lahore 54000, Pakistan

**Keywords:** deep learning, machine learning, diabetic retinopathy, medical imaging, color fundus images, image processing, image recognition, computer vision, segmentation, classification

## Abstract

Deep learning (DL) enables the creation of computational models comprising multiple processing layers that learn data representations at multiple levels of abstraction. In the recent past, the use of deep learning has been proliferating, yielding promising results in applications across a growing number of fields, most notably in image processing, medical image analysis, data analysis, and bioinformatics. DL algorithms have also had a significant positive impact through yielding improvements in screening, recognition, segmentation, prediction, and classification applications across different domains of healthcare, such as those concerning the abdomen, cardiac, pathology, and retina. Given the extensive body of recent scientific contributions in this discipline, a comprehensive review of deep learning developments in the domain of diabetic retinopathy (DR) analysis, viz., screening, segmentation, prediction, classification, and validation, is presented here. A critical analysis of the relevant reported techniques is carried out, and the associated advantages and limitations highlighted, culminating in the identification of research gaps and future challenges that help to inform the research community to develop more efficient, robust, and accurate DL models for the various challenges in the monitoring and diagnosis of DR.

## 1. Introduction

**Figure 1:**
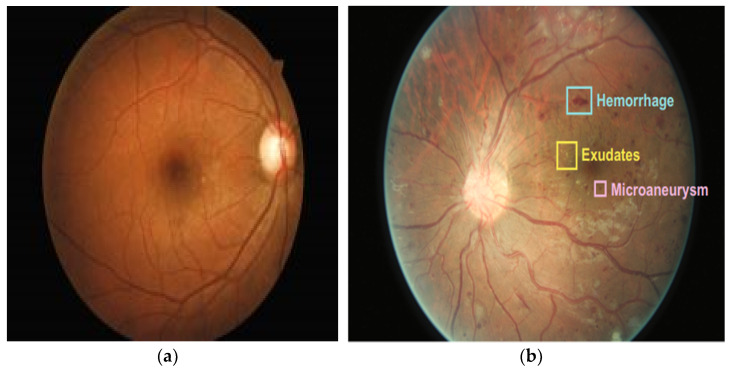
(**a**) Eye structure of Non-DR patient; (**b**) Eye structure of DR patient.

Diabetic Retinopathy (DR) is a harmful disease and the main cause of blindness among the working-age population. Moreover, DR is the most feared complication of diabetes and increases the chance of the onset of other diseases, such as kidney disorders [1], heart disease [2], and mortality [3]. The onset and progression of DR are most significantly associated with three risk factors: an increase in blood pressure, poor glycemic control, and long periods in a diabetic condition [4]. Figure 1 shows the eye structure of a healthy person and that of a DR patient.

According to World Health Organization (WHO) [5], 422 million people suffer from DR, with the number of patients expected to reach epidemic levels worldwide in the next few decades. In 2017, ~425 million DR patients were reported globally, estimated to reach 642 million by 2040. Patients with diabetes type-1 mellitus and ~60% of patients with diabetes type-2 mellitus will develop DR within 20 years post the onset of the condition [6]. DR is a costly disease. According to the national study AusDiab, the total direct cost owing to the treatment of DR in Australia was AUS$ 4.8 billion over ≥30 years [7]. In summary, DR has significant public health, clinical, and economic consequences.

Traditionally, color fundus photographs (CFPs) have been used for the analysis of DR, executed by a trained grader or retina specialist. Due to the low adherence and access to retina screening, a large proportion of undiagnosed patients have not received timely and appropriate medical support [8,9], the result being the continual increase of the DR population towards pandemic levels [10,11,12]. The early detection of the onset and progression of the disease are central to the mitigation of the threat of DR, allowing time to render the most impactful treatment before reaching criticality.

The risk of blindness can be reduced markedly through evidence-based treatment, with clinical studies showing that a reduction of over 90% of the risk is achievable [13]. For example, laser photocoagulation has been proven to be the most effective technique for the treatment of early-stage DR, which is core to the prevention of vision loss. However, patients with diabetes are not sufficiently aware, nor do they have knowledge, of DR. The rate of awareness is highly dependent on the region, country, and the duration of the diabetes condition, but, in general, it is low globally, e.g., the rates were 65%, 27%, and 42–60% in the USA, India, and Pakistan, respectively [14,15]. Additionally, at the early stage of the onset, visual symptoms are negligible (or indeed non-existent), a major factor that impedes awareness. As a consequence, the WHO recommends eye examinations for diabetes patients annually, as timely and accurate analysis based on relevant data helps to reduce the economic burden on health service providers. A recent study in Germany evaluated the cost as a function of the progression of DR [16], showing that the early non-proliferative management of DR is significantly more cost-effective compared to the management of proliferative DR.

The prevalence of DR has been increasing consistently over the recent years in both developing and developed countries. Moreover, the implementation of annual eye examinations is challenging in remote or rural geographies due to limited access to trained ophthalmologists. Specific programs are required to target these regions; otherwise, the successful treatment of DR will remain compromised. The USA and Europe have implemented DR management programs, examples being the USA 2000 diabetes program [17], and the France [18] and UK [19] DR screening programs. Most programs are founded on the use of CFIs for the analysis of DR. A specialist examines the images and assigns grades. On the whole, the successful delivery of the programs not only increases the workload burden on ophthalmologists, but also increases the cost of the analysis and treatment. The combination of limited access to experienced ophthalmologists coupled with the growing number of patients inevitably results in a prohibitive overall burden for existing healthcare systems. Consequently, the scope of these challenges has motivated extensive research and the development of techniques that provide a decision support capability for the analysis and early identification of the onset of DR automatically, primarily driven by the need to reduce the cost of the management of the condition [20].

Advancements in medical technologies have supported the universal goal of optimizing the efficiency of healthcare systems [21,22]. For example, e-health systems are being used successfully in a number of healthcare pathways [23,24]. Computer vision-based applications are gaining more importance in the field of biomedical imaging, providing decision support information of value to the radiologist that enhances the diagnosis and better informs medical staff on the most effective treatments for key medical conditions. In the specific application domain of medical imaging, different image modalities, such as color fundus images (CFIs), 7-field color fundus photographs (CFPs), and ultra-wide-field scanning laser ophthalmoscope (UWF-SLO), have been used for the analysis and treatment of DR.

Several approaches based on hand-engineered features have been shown to yield effective results in the recognition of the signs of DR in the regions within retinal fundus images. A number of traditional machine learning models using hand-engineered features for the analysis of DR have been reported [25,26,27,28]. For example, in [25,28], the performance of the diagnosis of DR was compared as a function of a number of different methodologies, such as mathematic morphology, thresh-holding and deformable models, retinal lesion tracking, matched filter models, clustering-based models, and hybrid approaches. In [26], the authors presented an overview of the algorithms that extract lesions from color fundus images, including features such as hemorrhages, blood vessel texture, and micro-aneurysms. The research related to exudate detection has been reviewed in [27], and an overview related to the segmentation of retinal vessels algorithms has been presented in [29]. Furthermore, [30,31] reviewed the different methods for optic disc segmentation and the analysis of glaucoma. However, expert knowledge is necessary in order to select the most appropriate hand-engineered features and, thus, these techniques are not generalized.

In recent years, a large body of research targeting the development of deep learning applications in e-healthcare has been reported, fueled by the ready availability of appropriate (large) data sets and low-cost access to computing resources [32,33,34]. DL-based solutions have been shown to offer outstanding performance for a range of computer vision tasks, and superior performance with respect to traditional, manual techniques. Similarly, a large number of DL-based models and algorithms have also been developed to analyze retinal fundus images as part of the goal to develop automatic computer-aided decision support systems that aid in the diagnosis of DR. DL-based applications for the processing of medical images have been developed and tested to extract DR-related signs. Several computer-aided systems that combine advanced algorithms and telemedicine technologies have been proposed for the early identification of the onset of DR and have been evaluated within DR screening programs. An automatic DR grading system provides the early detection and analysis of the DR, thereby triggering a referral to an ophthalmologist. The value proposition of these systems is the reduction in the workload of the ophthalmologists and, in turn, an increase in the cost-effectiveness of the analysis and treatment.

**Figure 2:**
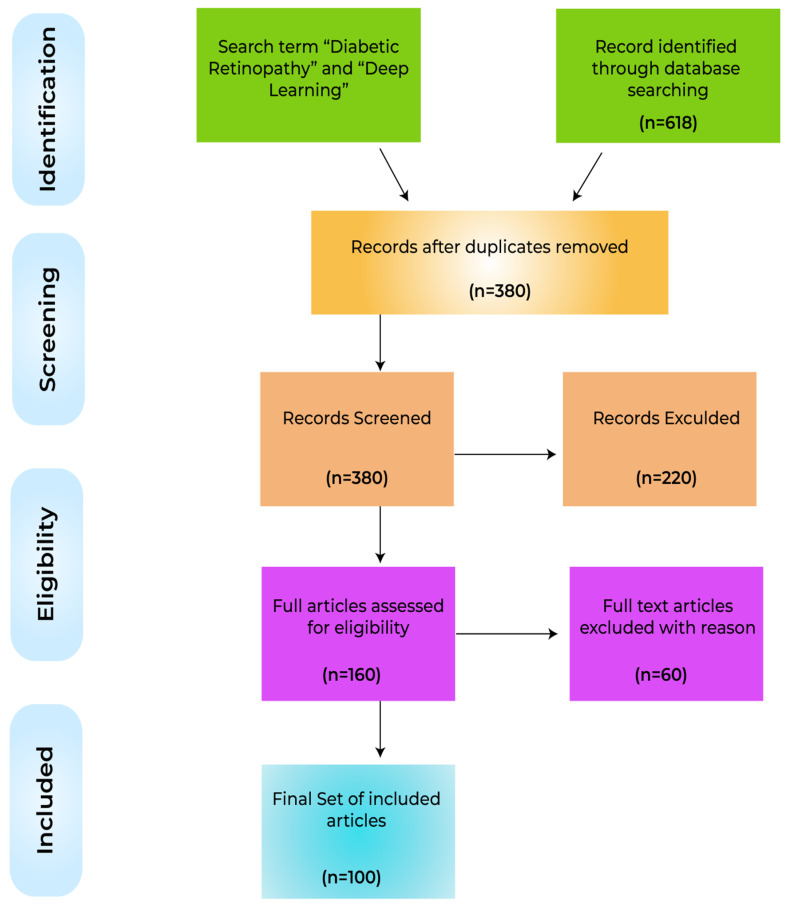
Flow diagram of the PRISMA approach used in the execution of the review.

**Figure 3:**
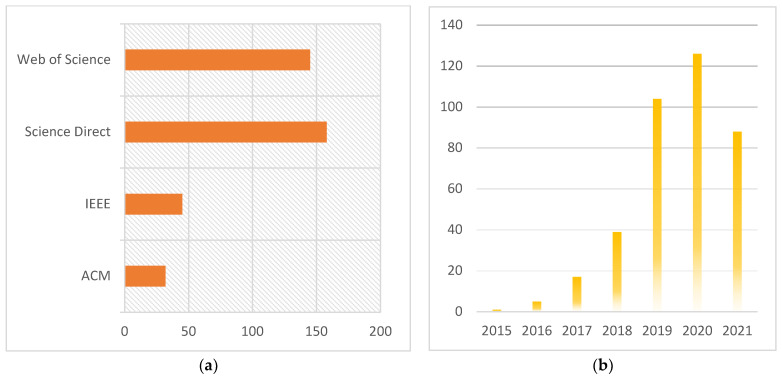
(**a**) Breakdown of recent research publications on DR analysis using deep learning in different databases; (**b**) Year-wise breakdown of recent research publications on DR analysis using deep learning.

Given the extensive body of recent research, a wide-ranging review of the advancements in the state-of-the-art techniques for the application of deep learning to the analysis of diabetic retinopathy is timely. This review distinguishes the key tasks inherent to the analysis of DR, specifically the retinal blood vessel segmentation, the prediction and identification, the recognition and classification in both applications, and methodology-driven perspectives. Reported deep learning-based models/algorithms, techniques, methodologies, systems, architectures, frameworks, and clinical studies for DR analysis are considered together, with an overview of the feature extraction techniques, tools, data sets, languages, and libraries used for the development of recognition, simulation, and evaluation frameworks. The review culminates with the identification of the research challenges that remain, such as open issues related to the definition and establishment of monitoring and treatment regimens for patients. The review adopts the PRISMA approach, illustrated in the form of a flow diagram depicted in Figure 2. Relevant research has been reported in a range of journals and conferences, and the breakdown of the body of research related to DR analysis using deep learning is captured in Figure 3, showing the total number and the yearly growth in the number of publications in different databases. Recent publications are presented in tabular form in an effort to assist readers in gaining an overview of the field simply.

A large number of predominately peer-reviewed publications reporting on an extensive variety of deep learning applications for the analysis of DR have been considered. The (“Diabetic Retinopathy” AND “Deep Learning”) query was performed regarding the title and abstract for the identification and selection of the most relevant publications. In summary, this review provides:

- A wide-ranging overview of the state-of-the-art techniques for deep learning development in the field of diabetic retinopathy that will help to inform the research community on future research in this domain.
- A description of the different tasks inherent to the analysis of DR, including retinal blood vessel segmentation, prediction and identification, and recognition and classification. Also provided are the most appropriate datasets aligned to the need to develop algorithms for DR analysis.
- Extensive bibliographic reference sources on deep learning algorithmic research for the analysis of DR.
- Deep learning-based algorithms, methods, models, architectures, systems, frameworks, and approaches for the analysis of DR are considered
- The most successful DL-enabled solutions for DR analysis are highlighted
- The performances of reported techniques are compared, research gaps are identified, and the future evolution of the application of deep learning for DR analysis is addressed.

## 2. Deep Learning for the Analysis of Diabetic Retinopathy

DR analysis is segmented according to established clinical practices into five categories: (i) screening and recognition, (ii) retinal blood vessel segmentation, (iii) lesion detection, (iv) lesion classification, and (v) validation. The state-of-the-art deep learning development for each category is presented.

### 2.1. Screening and Recognition

The quality of fundus retinal images has a significant impact on the development and, in turn, the performance of DL models for the analysis of DR. The automatic detection of image quality during the acquisition process has been proven to enhance the performance of models.

VariSeeTM is a deep learning-based software proposed for the screening of DR [35]. A convolutional neural network (CNN) was used in the learning stage of VariSeeTM, with the training comprising two phases: pre- and fine-tune training. In the first phase, an Inception-V4 network architecture, proven to yield the best performance in the screening of referable and of any DR, was used to speed up the training process. The CNN was trained using 31,612 color fundus images. The base model was then trained using 5649 color fundus images on the same network architecture during the fine-tune phase. Finally, to further improve the screening performance, the combination of two different Inception-V4 networks were deployed. The CNN model with Inception-V3 based software was developed for the automatic screening of DR, as reported in [36], and is founded on the detection of either the absence or presence of referable DR and non-DR. The algorithm was trained and evaluated using the EyePACS and MESSIDOR datasets.

A DL Inception-V3 model with transfer learning, which used 7-field color fundus photographs (7F-CFP), was proposed in [37] for the automatic screening of severe non-proliferative DR (NPDR). The model operates at the image level for all seven fields of view, according to the diabetic retinopathy severity scale (DRSS), with the prediction at the eye level, performed through the average predictions across all fields view of.

The method presented in [38] targets the determination of the quality of fundus images during acquisition. A deep CNN (DCNN) architecture consisting of five convolutional and two fully connected layers, along with a binary classification layer, were trained to grade the image quality automatically. The first convolutional layer of the DCNN comprised 96 filters of size 11 × 11, the second layer 256 filters, the third and fourth layers 384 filters, and the last layer 256 filters. The activation size of the first and final fully connected layers consisted of 4096 filters. Thus, the final output fully connected layer produced a 4096-dimensional image.

Publicly available data sets offer a limited number of fundus images, which in turn compromises the performance of the models. However, the authors of [39] detailed a data set consisting of 13,673 fundus images collected from 9598 patients. The images were divided into six different classes through seven graders according to quality, resulting in a data set entitled DDR, an enabler of the development of algorithms for the classification, detection, and semantic segmentation of DR lesions. A range of well-known DL algorithms, such as GoogleNet [40], ResNet-18 [41], VGG-16 [42] SE-BN-Inception [43], and DenseNet-121 [44], have been evaluated using the DDR data set for DR grading.

A deep visual features (DVFs)-based approach was proposed in [45] to automatically grade the severity level of DR (SLDR). The system does not need to perform pre- nor post-processing on images and learns through DVFs. The gradient location orientation histogram (GLOH) [46] and DColor-SIFT [47] techniques are used for the extraction of the DVFs. The latter method describes the color content and color variations of the retinal image. The GLOH feature method is used to improve the classification performance, and principal component analysis, PCA, together with GLOH, is used to reduce the size of the descriptor. The mathematical formulation of these methods is as follows:

(1) $$h\left(x,y\right)=arglog-polar\overset{n}{\underset{k=0}{\sum }}R_{g}{\left(x,y\right)}^{k}\;G\_H\left(x,y\right)=arg\;max_{PCA}\left[h\left(x,y\right)\right]\;$$

A min–max scaling approach is then used to combine the features of both DColor-SIFT ad GLOH. Equation (2) is the visual feature vector constituted by the deep learning neural network (DLNN) algorithm in the creation of the DVFs:

(2) $$f\left(x,y\right)=arg_{min-max}\left\{h\left(x,y\right),G\_H\left(x,y\right)\;\right\}\;$$

A new compression layer and fine-tuning steps were added to a development deep learning NN (DLNN) framework in order to learn the most appropriate DVFs features. The DLNN consisted of three active layers, viz., the base, compression, and prediction layers. Restricted Boltzmann machines (RBMs) [48] were deployed at the base layer to increase the unsupervised power for the learning of features. The compression layer was generated with the aid of Shannon entropy constraints (SECs) [49] that compute the effective weights, unsupervised, using the output of the base layer, thus refining the weights of the features. Finally, the linear soft-max classifier [50] was used in a supervised manner within the prediction layer to output the final label for the DR. A comparison of the performance of the abovementioned methods is given in Table 1.

### 2.2. Retinal Blood Vessel Segmentation

The early detection of changes in the fine retinal blood vessels is important, as pathological retinal damage causes vision impairment. However, blood vessel segmentation is a challenging task due to the low contrast of retinal images, the presence of pathologies, such as HMs and micro-aneurysms (MAs), and variations in morphology in noisy background images. State-of-the-art deep learning developments in the segmentation of retinal blood vessels are reviewed in the following section.

#### 2.2.1. Convolutional Neural Networks (CNNs)

A framework based on a contrast limited adaptive histogram equalization (CLAHE) was proposed for the segmentation of morbid retinal images in [51]. The approach was successful in the elimination of the background from the input image and in enhancing the pixels of blood vessels in the foreground. Furthermore, evidence was provided to show that the tandem pulse coupled neural network (TPCNN) model is effective in the automatic generation of feature vectors. Finally, a DL-based support vector machine (DLSVM) model has been shown to predict and identify the class of the blood vessels. The firefly algorithm is used for the fine-tuning of the DLSVM parameters. The mathematical representation of the firefly is given in Equation (3):

(3) $$dist_{ij}=\frac{\left\|x_{i}-x_{j}^{k+1}\right\|}{\sqrt{\sum_{l=1}^{n}{\left(x_{il}-x_{jl}^{k+!}\right)}^{2}}}\;$$

where $x_{i}$ and $x_{j}^{k+1}$ represent the number of firefly swarms, and $x_{il}$ and $x_{jl}^{k+!}$ represent the brightness of the corresponding fireflies. $dist_{ij}$ is the distance vector between the attractiveness of the fireflies and l is the candidate index of the fireflies. Equation (3) indicates that the attractiveness between$\;x_{i}$ and $x_{j}^{k+1}$ is proportional to the firefly.

A total of 12 CNN models have been employed [52] for the segmentation of the non-vessel and vessel pixels. Every model consists of two fully connected and three convolutional layers. The DRIVE dataset was used for the evaluation of the method. Maninis et al. [53] used a pre-trained visual geometry model (VGG-net) for the image-to-image level segmentation of the blood vessels. The model was modified by removing the fully connected layers, with the extra convolutional layers added after the first four convolutional blocks of VGG, before the pooling layers. STARE and DRIVE datasets were used for the evaluation.

Wu et al. [54] reported the first use of a CNN for the extraction of the discriminative features, a PCA-based nearest neighbor search for the estimation of the local structure distribution, and a generalized probabilistic framework for the segmentation. The DRIVE dataset was used for the evaluation. A seven-layer CNN model that simultaneously segments the blood vessels, fovea, and optical discs (OD) was proposed by Tan et al. [55]. After the normalization and pre-processing of the images, a classification problem was formulated rather than carrying out a segmentation. Assuming the four classes for the blood vessels, fovea, OD, and background, 25 × 25 neighborhood pixels were used for each pixel classification. This model is complex and time consuming, as every pixel is classified independently.

A fully convolutional network (FCN) in tandem with structured prediction was used for segmentation, the task being formulated as a multi-label inference [56]. The green channel of the color fundus images was pre-processed by contrast, normalization, gamma adjustment, and intensity scaling between 0 and 1. The DRIVE dataset was used for the evaluation of the method. Fu et al. [57] formulated the segmentation task as a boundary-detection task and proposed a method utilizing the fusion of the fully connected conditional random field (FCCRF) and FCN. First, the FCN was used to generate the probability maps of the blood vessels, and then FCCRF was used to combine these probability maps with long-range pixels for the segmentation. In [58], the authors used an FCN incorporated with some auxiliary classifiers at intermediate layers to make the features more discriminative at the lower layers. Transfer learning was used to train the FCN model in order to overcome the limited number of samples available.

In [59], a deep CNN-based method for the pixel-wise supervised vessel segmentation was proposed. The model was trained using color fundus images that were pre-processed with zero-phase whitening and contrast normalization, and gamma corrections and a geometric transformation process were used for the augmentation. The evaluation of the model was executed using STARE, DRIVE, and CHASE data sets. The performance results indicate that the model is sensitive with respect to the detection of fine vessels and robust against the central vessel reflex.

A principle component analysis (PCA) approach has been used to improve the picture splendor and contrast for the segmentation of the eye veins [60]. This technique performs the vein division and is known as tale solo calculation. The standard cut division chart along the curvelet change is used to segment the thickness of the vein, with a multi-goal curvelet change supporting the division of the veins. The PCA calculation provides the picture’s slope for the vein division. In the retinal images, the optic circle is a critical component for the occurrence of DR. This technique also uses Hough change, which helps one to recognize the elliptic state and roundabout of the optic plate and focus on the ROI that contains it. The adjusted assumption amplification calculation is used for the fragments of hard exudates from the fundus image. The bandlet change and gray level co-occurrence matrix (GLCM) are used for the computation of the highlight arrangements in the image. Finally, the CNN segments the highlighted regions of the image for DR. Another DL system for the identification and detection of proliferative-stage diabetic retinopathy (PDR) was proposed in [61]. The hallmark features can also be used for the detection of PDR, and this is called neovascularization. The aim of the system is to correctly detect the presence of neovascularization using color fundus images. If the neovascularization is found in the eye, this means that the eye is affected by PDR. Neovascularization is also known as the formation of new abnormal blood vessels in the retina. Thus, the formation of neovascularization may lead to partial or complete vision loss. This system is based on CNN, which is modeled with VGG-16 Net architecture and segments the abnormal vessels of the retina. DRIVE and STARE datasets were used for the evaluation of the system.

#### 2.2.2. Stacked Auto Encoder (SAE)

A hybrid DL architecture, which consists of unsupervised stacked de-noising autoencoders (SDAEs), was proposed by Maji et al. [62] for the segmentation of blood vessels in the fundus images. The structure of the architecture has two DAEs; the first DAE has 400 hidden neurons, and the second DAE consists of 100 hidden neurons. The RF is used for the classification of features and, after that, SDAE learns those features. In this approach, a patch of size k ± k is used around each pixel in the green channel for the segmentation of the vessels. The authors used the DRIVE dataset for the evaluation of their architecture.

Roy and Sheet [63] proposed a stacked auto-encoder (SAE)-based deep neural network (DNN) model for the segmentation. For the training of this model, the domain adaptation (DA) approach was used. This model consists of two hidden layers, and supervised learning and auto-encoding mechanism are used for the training of these layers. After that, the DA is applied in two stages: supervised fine-tuning and unsupervised weight adaptation. In the unsupervised weight adaptation stage, the unlabeled samples from the target domain are used with the auto-encoding mechanism and node dropouts for the re-training of the hidden nodes. In the supervised fine-tuning stage, a small number of labeled samples from the target domain are used for the fine-tuning of the SAE-DNN. The results show that the performance of the SAE-DNN model can be increased by using DA domain.

A supervised DL-based approach that segments the blood vessels from the green channel was proposed by Li et al. [64]. This approach labels the pixel in a patch form instead of a single pixel. In this approach, the DNN, which consists of DAEs, is used to transfer the retinal image to a vessel map for the segmentation.

A two-level ensemble of SDAEs was proposed in [65]. In the first level of the ensemble, N number of SDAEs are composed to form a same structure network (E-net). Each SDAE has two hidden layers and a SoftMax classifier. Bootstrap training samples are used to train the SDAEs, which is followed by auto-encoding mechanism. After that, the fusion strategy is used to combine different SDAEs and this produces probabilistic maps of images. In the second level of the ensemble, the convex weight average (CWA) is used to merge the decisions from the two different e-nets, which have different architectures, to further explore the diversity for the segmentation.

Fu et al. [66] formulated the blood vessel segmentation problem as a boundary detection task and proposed a deep-vessel segmentation method by integrating conditional random field (CRF) and CNN as a recurrent neural network (RNN). A summary of the reported techniques for retinal blood vessel segmentation is given in Table 2.

### 2.3. Detection

#### 2.3.1. Convolutional Neural Networks

An automatic CNN-based DR grading system [67] has been reported for the classification of retinal images into four severity levels. The CNN combines the input images through an appropriate weight matrix to extract the specific features of the images without losing the spatial arrangement information. Another automatic DL-based model for the detection of DR severity that also includes pre-processing, recognition, and detection is presented in [68]. Blood vessel extraction, the green channel extraction, and the optic disc (OD) removal are performed at the pre-processing stage, and the green channel extraction enhances the contrast of the images. A morphological operation removes the OD and the kernel fuzzy c-means method is used for the extraction of the blood vessels. The recognition of DR features is achieved at the second stage. The recursive region growing segmentation (RRGS) algorithm is used to recognize the hard exudates; a Laplacian–Gaussian Filter (LGF) and matched filtering and mutual information are applied for the recognition of micro-aneurysms (MA) and hemorrhages (HEM); and the extracted features, such as the MAs counts, area and exudate counts, perimeter, and the area and perimeter of the blood vessels are then inputted into the CNN for detection.

The CNN-based automatic diabetic detection model for retinal images was presented in [69] and consists of five modules, including pre-processing, exudates segmentation, blood vessel segmentation, texture features extraction, and DR detection. In the pre-processing step, adaptive histogram equalization (AHE) is used to enhance the quality of the input retinal images. In the second step, the tasks of exudate and blood vessel segmentation are performed by fuzzy c-means clustering (FCM) and CNN, respectively. The texture features are then extracted from the exudates and blood vessels, followed by a support vector machine (SVM) implementation for the detection of DR.

A hybrid approach consisting of CNN and a linear support vector machine (LSVM) was presented in [70]. The CNN model extracts the features from the input fundus images, and these features are subsequently inputted into the to LSVM for binary classification as DR or non-DR. In another study [67], the authors concluded that most errors occurred in the misclassification of the mild disease as normal, as the developed CNN model was not capable of detecting the subtler features of the disease. As a consequence, the contrast limited adaptive histogram equalization (CLAHE) was used to ensure the fidelity of the dataset for the verification of the class labels and, in turn, to enhance the model’s capability to recognize subtle features. These subtle features were then inputted into pre-trained AlexNet and GoogleNet models for the final detection of DR. Another hybrid model fuses the Inception-ResNet-v2 and CNN models [71]. The former, trained on the ImageNet dataset, is used for transfer learning, whilst the latter is a customized convolution block followed by fully connected layers. This approach removes the last layers of the Inception-ResNet-v2 and adds the CNN custom block followed by fully connected layers, global max-pooling, and SoftMax.

A CNN-based method consisting of seven blocks of two layers accepting 3 × 640 × 640 images was presented in [72]. The size of the images was progressively reduced until a receptive field of 64 × 5 × 5 was attained for the extraction of features. Every layer had a stack of 3 × 3 convolutions, along with a 1 × 1 stride and 1 × 1 padding, followed by batch normalization and ReLU activation function. The final vector had the 64 values derived from 4 × 4 average pooling layers. At the final layer, a linear classifier and a SoftMax function operated on the 64 features for the grading and detection of DR, according to the international clinical diabetic retinopathy severity scale (ICDR) [22]. The quadratic weighted kappa (QWK) was used as a loss function for the optimization of the CNN parameters. Another DL system consisting of three CNNs (ResNet-50) and relying on transfer learning was presented in [73], using data acquired from ultra-wide-field scan laser images instead of fundus images for the training. An ensemble of an orthogonal learning particle swarm optimization (OLPSO)-based model and CNN model (OLPSO-CNN) consisting of three main processes, including pre-processing, feature extraction, and detection, was presented in [74]. The noise of the input image was removed at the pre-processing stage, followed by the segmentation of the pre-processed image using the watershed algorithm. The OLPSO-CNN then extracted the features from the segmented image, and these extracted feature vectors were fed into the DT for classification. The MESSIDOR dataset was used for the evaluation of the performance of the method. A DL model consisting of different pre-trained CNN architectures, coupled with transfer learning and hyper parameter tuning treating different imbalanced classes of retinal images, was proposed in [75]. The model gives better results when presented with imbalance data.

DR is also characterized by features such as bright lesions, red lesions, and neovascularization. The former are clinically observable lesions occurring after the appearance of red lesions. Also important to the diagnosis are the exudates (hard and soft exudates) and cotton wool spots. A CNN-based DL framework that detects bright lesions was proposed in [76]. At the pre-processing stage, the background removal of images, OD elimination, and segmentation of candidate lesions were performed, with the segmented bright lesions fed into a CNN for detection. The MESSIDOR dataset was used for the evaluation of the framework. A modified version of CNN with a standard VGG-16 network was proposed [77]. An originally trained VGG network on the image-net dataset was used for the modification of the CNN, with transfer learning on VGG-16 improving the generalized capabilities of the CNN in DR detection.

A CNN independent adaptive kernel visualization technique introduced in [78] converts the original input image into smaller sub-images by applying a sliding window sized 28 × 28 pixels with a stride of 3 pixels, thereby producing a 172 × 172 (i.e., [(544 − 28)/3] × [(544 − 28)/3)]) features map. The sub-images were also useful for the further training of the model. The model also has a threshold adjustment scale to achieve the optimal heat maps. In [79], the performance of the CNN was evaluated using the original fundus photographs and entropy images. The original images were transformed into entropy images using block size 9, following the scaling of the pixel value of the original image between 0 and 1, from 0 and 255, creating downsized images with a standard resolution of 100 × 100 pixels. The spatial entropy is a function used to compute the probability distribution for the local gray values. The local entropy for the original image can be computed as follows (Equation (4)):

(4) $$E_{local}=-\sum_{i}P\left(i\right)\times log_{2}P\left(i\right)\;$$

where P(i) represents the relative frequency of the i-th gray level of an n × n block. The statistical characteristics of the local regions generated by local entropy were used to learn the local structural information about the image [80]. Finally, the entropy images were fed into a CNN architecture for feature extraction and detection.

Multiple instance learning (MIL) techniques have been proven to yield improved performance compared to supervised learning approaches. Image-level annotation is only needed for the detection of lesions. However, these techniques do not exhibit acceptable performance with respect to hand-crafted features. As a consequence, a deep MIL-based detection model that jointly learns the features and classifiers to improve the detection performance for DR images and their lesions was proposed in [81]. Furthermore, a pre-trained CNN network was used for the estimation of the patch-level DR prior to the application of a global aggregation to detect the images. An end-to-end multi-scale scheme was also proposed to better treat irregular lesions.

A trilogy of skip-connection deep networks (Tri-SDN) architecture was proposed [82] for the identification of the relationship between the baseline, the follow-up information on the retinal fundus images, and the electronic medical record (EMR)-based attributes. The architecture also extracts valuable clinical information, along with the aforementioned systemic attributes from fundus images. The architecture comprises: (i) a CNN followed by the global average pooling (GAP) and a subsequent DNN with skip-connection blocks (SCB) that encode the salient features of the lesions existing in both the follow-up and baseline images; (ii) SDN extracts the latent features and shows an inter-relationship between the systemic attributes of interest, as well as the intra-relationship between the follow-up and baseline values of each parameter which exists in the EMR; and (iii) another SDN that classifies the risk of DR progression through the concatenation of EMR-based and fundus-based features. The skip-connection blocks are the key components of the Tri-SDN, rendering the end-to-end flow of the signals more efficient during the feed-forward and back-propagation processes. An AlexNet DNN-based computer-aided diagnosis (CAD) system has been applied for the optimal identification of DR [83]. The method is founded on the CNN model and consists of modules that included pre-processing, segmentation, feature extraction, and classification. The Gaussian mixture model (GMM) and adaptive learning (AL) was used for the segmentation. Connected component analysis was used for the localization of the region of interest (RoI). The AlexNet-based DNN model was used for the extraction of the high dimensional features, the selection of which was performed by linear discriminant analysis (LDA) and principle component analysis (PCA). An SVM was used for the optimal classification.

A DL algorithm which quantifies the non-perfusion area (NPA) on montaged wide-field OCT angiographies (OCTA) through the segmentation of the NPA at three different locations, including the macular, nasal, and temporal scans, for the assessment of the DR severity was detailed in [84]. A residual module from ResNet improved the training of the model, yielding faster convergence and higher accuracy using identity short cut connections [85]. The U-Net architecture was also used for the backbone of CNN, providing several adaptions for the detection of NPA in wide-field images. The OCT reflectance images of the inner retina and the inner retinal thickness were combined and fed into a subnet to reduce the computational complexity. A subnet segmented the shadow artifact-affected areas of the images, and another subnet extracted the retinal capillary features from the face images. The subnet outputs for the vessel and shadow detection were then inputted into three parallel subnets that learned the features from these three regions (macular, nasal, and temporal). Finally, the results of these three parallel subnets were concatenated for the assessment of the true and the artifacts shadow-affected NPA area.

A number of CNN-based models were explored in [86]. The results showed that an Inception-V3 model provided better results as compared to traditional CNN models. Multiple filter sizes at the same level, label smoothing, RMS-Prop batch normalization factoring, and dimensionality reduction were then used to improve the performance further. Inception ResNet-V2, a combination of ResNet and Inception-V4, was developed to exploit deep residual learning. The ResNet-V2 contains hybrid inception modules, in which residual connections add the output of the convolutional operation of the module to its input.

A validation of the commercially available RetCAD v1.3.0 system that executes the joint automatic detection of age-related macular degeneration (AMD) and DR was presented in [87]. The color fundus image was the input to the system, executing a conversion into RGB and contrast-enhanced (CE) images. The inner structure was composed of two ensembles based on three CNN architectures (CNN1, CNN2, CNN3), followed by multiple convolutional blocks, and pooling and dense block layers. Moreover, each ensemble consists of six other CNNs, in which an RGB image was inputted into three CNNs, and a CE image was input to the other three. A final score between 0 to 100 was computed by the average of all the scores generated by the networks in each ensemble.

#### 2.3.2. Deep Convolutional Neural Networks (DCNN)

A deep convolutional neural network (DCNN)-based model trained and tested using a retrospective development dataset consisting of 1,28,175 retinal images graded between three to seven times was reported in [88]. An ensembles approach that extracted rich features from the retinal image to improve the detection accuracy consisted of five deep CNN models that included Inception-V3 [89], Resnet50 [90], Dense-121 [44], Dense-169 [44], and X-ception [91] was proposed in [92]. The iterative optimization (fine tuning) of the CNN models reduced the empirical loss, formulated as (Equation (5)):

(5) $$L\left(w,\;X_{i}\right)=\frac{1}{n}\underset{x\in X_{i},y\in Y_{i}}{\sum }l\left(h\left(x,w\right),y\right)\;$$

where h(x,w) represents the CNN model, x is the input, y is the predicted class given by w, and l is the categorical cross-entropy loss penalty function.

The authors of [93] harnessed ultra-wide-field images [93,94,95] that capture up to 82% of the retinal surface, as compared to the convention fundus images, to develop a detection system. The system segmented the region of interest (RoI) to remove undesirable components, such as skin and eyelashes, using the residual network, which consisted of 34 layers (ResNet-34). A deep DR system, able to provide the detection of early- to late-stage DR was described in [96]. The system comprised three DL subnetworks (all consisted of ResNet [41] and Mask-RCNN [97]), including image quality assessment, lesion-ware assessment, and DR grading. The image quality assessment subnetwork executed a binary classification of the image, viz., a determination of whether the image is recognizable and gradable by assessing the clarity, artifacts, and other problems of the retinal images; the lesion-aware subnetwork, used to label retinal lesions for the segmentation and detection of hard exudates, micro-aneurysms, and hemorrhages; and the grading subnetwork, the fine-tuning of which was performed on a pre-trained ImageNet network. Finally, the lesion features extracted by the lesion-aware subnetwork and features extracted by the grading subnetwork were concatenated to improve the grading performance of the system.

The densely connected convolutional network (DenseNet-169) proposed in [98] assigned weights to the entire network instead of solely assigning them to the last (or top) layer. The last layer was designed using global average pooling 2D (GAP-2D), along with a 0.5 value set dropout layer. GAP-2D considers the whole input block as a pool size, with the dropout layer resolving the issue of over-fitting. The Adam optimization algorithm (AOA) was used to optimize the weights of the model, a sequential modelling methodology used for customizing and adding more layers, such as the dropout, convolutional, optimizers, and dense. The authors of [99] introduced the Inception-V3 based architecture, which applies multiple convolution filters to the input image. At the same time, a pooling process is initiated, followed by a concatenation of all the generated results. This architecture has the capability to extract multiple features from the same input image for classification.

An ultra-wide-field fundus image-based deep convolution neural network (DCNN) [100] using a VGG-16 DCNN to learn the local features of the image and generate a detection was detailed in [101,102]. The model resized the original input retinal images of an aspect ratio of 3900 ×  3072 pixels to create images of 256  × 192 pixels. The resized images were fed into the VGG-16, which consisted of five blocks with two fully connected layers.

Another study [103] presented a deep learning system (DLS) which used the low fraction of high-resolution images for the training. Each retinal image was graded into three categories, viz., the macular edema, diabetic retinopathy, and grade-ability. The image grade ability was examined further by the two stage-system that determined whether the image is gradable or not. A DCNN was used for the extraction of the features from the color fundus images, with the Inception-V3 architecture used for the prediction of the class or grade of the retinal image. The network accepted 2095 × 2095 pixel input images for the training, with a mini-batch size of 1. The batch normalization layers were replaced with instance normalization layers, with the weights of the parameters updated by the accumulation of the 15 mini-batches.

A data-driven deep learning algorithm, the features of which—along with their metadata—were extracted from color fundus images, was reported in [104]. These deep features were fed into a tree-based model to obtain the final classification. The 75,135 color fundus images from the EyePACS dataset were used for the training and testing of the model. Image scaling was performed in the range from 0 through to 1, and the images were also converted to the standard resolution 512 × 512 pixels by cropping the inner retinal circle. Furthermore, the invariance between the color contrast of the images was encoded, and a brightness adjustment method was also proposed. The latter method adjusts the brightness of the images using a random scale α = [−0.3, 0.3] for each image, formulated as:

y=(x−mean)×(1+α) 

The contrast of the images was adjusted using a random scale β = [−0.2, 0.2], formulated as:

y=(x−mean)×(β) 

After pre-processing, the customized DCNN was used for the automatic learning of the deep features. The convolutional layer parameters of the network were used for learning and the filters were also used iteratively for the transformation of input images into hierarchical feature maps. The discriminative learning of the features depends on the spatial levels of the image and, thus, this obviated the need to tune the parameters manually. The convolutional layers were positioned successively, with the input image transformed at each layer, with the resultant output information propagated to the next layer. Deep residual learning (DRL) was used for the development of the custom convolutional network, the model being formulated as (Equation (6)):

(6) $$x_{l}=conv_{l}\left(x_{l-1}\right)+x_{l-1}\;$$

where $conv_{l}$ is the convolutional layer l, which returns the sum of both its output and the output of the previous convolutional layer. The summation of the convolutional layers facilitated the incremental learning for a polynomial function that, in turn, enhanced the characteristics of the retinal fundus image for the training of the DCNN, improving the overall performance of the identification model.

A two-stage model that used color fundus photographs (CFPs) was presented in [8] that aimed to predict the progression of the DR. At baseline, a set of seven CFPs images were given as inputs to the first-stage DCNNs, trained for each type of CFP field to establish the pillars. Random forest (RF) models then combined the probabilities of the individual pillars, and both the RFs and single pillars were trained to generate a binary outcome. The InceptionV3 architecture was used to create field-specific pillars, and a cascade transfer learning strategy was adopted to create the initial weights obtained by the training of ImageNet-40. The generated weights were also used to initialize the training of the pillars for the prediction of the DR progression. A DCNN-based system that analyzes micro-aneurysms in the fundus images was introduced in [105]. Maximum Gaussian–Laplacian (LoG) and mutual information (MI) filters were integrated for the identification of a range of lesions, regardless their scale, form, and texture. A band-pass Filter (BPF) was applied to enhance the contrast of the exudates after the lesions were extracted. A sparse principal component analysis (SPCA) was also deployed to obviate data imbalance issues.

#### 2.3.3. Deep Belief Networks (DBNs)

A framework which consisted of feature extraction and detection stages was proposed in [106]. In the former phase, input image features such as the local vector pattern (LVP), local binary pattern (LBP), and local tetra patterns (LTPs) were extracted. A deep belief network (DBN) then used those extracted features for the detection. In addition, the self-improved gray wolf optimization (SI-GWO) was applied as an activation function for the optimal tuning of hidden neurons in the network, which improved the overall accuracy of the framework. An automatic model which performed a number of tasks, including pre-processing, optical disk removal, blood vessel removal, abnormality segmentation, feature extraction, optimal feature selection, and detection, was detailed in [107]. The contrast limited adaptive histogram equalization (CLAHE) was used to pre-process the input image. The open-close watershed transformation was used for the removal of the optic disc, and the segmentation and removal of the blood vessels was performed using gray level threshold. Once the removal of the blood vessels and optic disc was completed, Gabor filtering and top-hat transformation were used for the segmentation of abnormalities. The feature extraction phase comprises four features, viz., the texture energy measurement, local binary pattern, and Kapur’s and Shannon’s entropy. The meta-heuristic algorithm, modified gear and steering-based rider optimization algorithm (MGS-ROA), was applied for the selection of the optimal features, also used to update the weight of the DBN. The selected features were fed into the DBN for detection.

#### 2.3.4. Transfer Learning

An Inception-V3 network deep transfer learning-based approach reported in [108] consisted of five convolutional layers, eleven inception modules, two max-pooling layers, one average pooling layer, and one fully connected layer that generated the image-wise categorization. Inception-V3 generates clusters of the same sparse nodes and positions them into a dense layer to increase both the width and length of the network and to reduce the computation burden efficiently. The associated known label and pixel intensities of each retinal image were fed into the network, and the features of the network automatically adjusted to provide an accurate detection. A transfer and ensemble learning-based technique proposed in [109] utilized pre-trained models, InceptionV3, X-ception, and Inception Resnet-v2. The IDRiD dataset was used for the evaluation of the performance.

A deep transfer learning (DTL)-based framework using optical coherence tomography (OCT) images presented in [110] consisted of 11 pre-trained DL models that included ResNet-18 [41], VGGNet16 [42], Google-Net [40], AlexNet [111], ResNet-50 [41], DenseNet-201 [44], InceptionV3 [89], Squeeze-Net [112], VGGNet-19 [113], ResNet-101 [41], and Inception-ResNet-v2 [85]. Amongst them, the training of the DenseNet-201 was optimized by freezing the layers of the network, performed by setting the learning rate to zero for all the initial layers. The weights of the frozen layers were not updated during the training of the network. The optimized DenseNet-201 was then used for the extraction of features that were core to the training of the ANN to accurately compute the classification. An Inception-V3 network, based on two versions of a DL system and aimed at patients with tele-retinal diabetic retinopathy in a primary care setting, was proposed [114] to support the monitoring and prediction of the likelihood of progression. In the first realization, the system operated on a primary field as the input (one field), and in the second implementation, nasal, primary, and temporal images were taken as the inputs (three fields). An identical Inception-V3 module with shared weights was used to process each field, and the classification layer concatenated the output features. Both versions operated using a color fundus image of 587 × 587 as the input and generated an output between 0 and 1 that indicated the likelihood for the development of the DR within 2 years. In [115], deep neural network-based GoogleNet, using manually modified Davis grading images, was proposed. This model used the retinal area of the images that is not typically visualized on the fundoscopy.

#### 2.3.5. Other Variants of Deep Learning Models

A multi-self-attention deep learning network proposed in [116] consisted of a feature extraction phase and a detection phase. The Inception-V3 model was used for the extraction of image features, automatically generating feature maps. The generated feature maps that replicate the condition of the retina were then used as an input to the network, which calculated the multi-self-attention features of the images. The connected stages and convolution layers detected the DR.

In another study [117], an optical coherence tomography (OCT) image-based automatic detection deep model, referred to as OCTD_Net, that generated a grade between 0 and 1 was presented. This detection model, known as OCTD_Net, consisted of two networks, Org_Net and Seg_Net, of which the former, Org_Net, used DenseNet blocks [44] integrated with squeeze-and-excitation blocks [43] for the extraction of the features from the OCT images, while the Seg_Net comprised a ReLayNet [118] layer, convolution block, and segmentation block used for the extraction of the features. The detection block combined the features extracted by both networks and added these bitwise to classify the OCT image as normal or as denoting the early onset of DR. The output of the system provided decision support, by indicating that grade-1 patients have significant changes in the thickness of the eye and certain reflections in the retinal layers, while patients with grade 0 do not have certain changes in eye. Furthermore, the model also provided evidence that patients with early DR exhibit different textures around the ellipsoid zones and the myoid, photoreceptor outer segments and the inner nuclear layers.

Another study that used optical coherence tomography angiography (OCT-A) images instead of color fundus images [119] compared the impacts of different feature engineering approaches on the detection performance of a DNN with unprocessed OCT-A images. The effect of a lower resolution on the detection was investigated and a generative adversarial network (GAN) was used to recover the lost features of the image. The relationship between the lateral resolution and the detection of the severity of DR was also explored.

A fully automated DL-aided framework, which detected vision-threatening and referable DR using the face structural, volumetric, and optical coherence tomography angiographic (A-OCT) data was proposed by Pengxiao et al. [120]. The framework used 3 × 3 macular image scans obtained from a spectral-domain OCTA system (Optovuelnc, Avanti RTVue-XR). The referable DR was graded at level 35 or worse, and the vision-threatening DR was graded at level 53 or worse, for any level of DME. The framework was constructed using 3D (EfficentNet-3D-Bo) and 2D (DcardNet-36). A deep neural network (DNN)-based algorithm that produced grades for retinal fundus images according to the international clinical diabetic retinopathy (ICDR) severity scale trained and validated in real time data that was reported in [121]. A low-cost system deep DR-Net amenable to implementation on a small embedded board was presented in [122]. At the core of the model was a cascaded classifier network encoder, integrated in a residual style to ensure an appropriately sized implementation. The different convolutional layers help to ensure the richness of network features for the grading of DR. A smartphone-based automatic model in [123] used models such as AlexNet, Google-Net, and ResNet-50 with transfer learning. The RestNet50 model was deployed in a smartphone-based model to explore the classification of DR using synthetic images, the major aim is to validate the performance of the DL models for a specific application. Table 3 summarizes the reported body of research on the detection of lesions.

### 2.4. Classification of the Lesions

The medical community has established a classification standard for DR based on four stages of severity [23], determined by the number and type of lesions (as exudates, micro-aneurysms, and hemorrhages) in the retina. Class 0 relates to no apparent retinopathy, class 1 to mild non-proliferative diabetic retinopathy (NPDR), class 2 to moderate NPDR, class 3 to severe NPDR, and class 4 to proliferative DR. The success of the deep learning techniques and methodologies in a range of applications has stimulated extensive research for the classification of diabetic retinopathy.

#### 2.4.1. Convolutional Neural Networks (CNNs)

A DL model comprising pre-processing and classification stages was proposed in [125]. Retinal images from different data sets were extracted to standardize their size in the pre-processing stage, and a CNN algorithm was used to generate the classification. In traditional methods, feature sets are created manually, but the proposed method executed the training phase of the DL models in a relatively rapid time using significant computing resources. An end-to-end CNN model was proposed in [126] for the grading of the severity of diabetic macula edema (DME). After cropping and re-sizing the image, the red, green, and blue channels were scaled to zero mean and unit variance. The model comprised three convolutional blocks and one block of fully connected layers, and the number of training samples was enhanced through data augmentation techniques.

Ting et al. [127] used the CNN model for the analysis of AMD and other DR complications, providing evidence that the proposed CNN was more effective compared to other reported models. However, the model was unable to identify all classes of DR complications using color fundus images. The classification model for AMD was trained using 72,610 images and tested on 35,948 fundus images from different ethnicities. A two-stage method consisting of a cascaded fully convolutional residual network (FCRN) with fused multi-level hierarchical information was reported in [128] to generate the segment axis and associated probability map. Pixels with maximum probability were then cropped from the segmented regions and fed into another residual network for classification.

An interpretable model based on a fully convolutional neural network (FCNN) that classified retinal images into severity levels and also provided additional information on the results of the classification by assigning a score to both the input and hidden layers in the network was proposed in [129]. The class was computed using the score of the pixel contributions, obtained by a pixel-wise propagation model that divided all neurons into scores. A score for each neuron was computed using Equation (7):

(7) $$S_{L}=\overset{L}{\underset{i=1}{\sum }}\left(\sum s_{ki}\right)+\left(\sum s_{Input}\right)\;$$

where $S_{L}$ is the score of the last layer, $s_{ki}$ represents the constant tensors for each layer, $\sum s_{ki}$ is used to compute the element-wise sum of the scores, and $\sum s_{Input}$ represents the sum of the pixel-wise scores of each input neuron. The generated visual maps support the ophthalmologist in the interpretation of the statistical regularities inherent to the classification.

A deep learning architecture that consisted of two modules, including a memory module and a central CNN, was proposed in [130]. The system firstly scanned and then pre-processed the fundus images of the eye. The maximal principal curvature (MPC) was applied to extract the branching blood vessels, using the maximum Eigenvalues of the Hessian matrix. Morphological opening and adaptive histogram equalization (AHE) were performed to eliminate and enhance the falsely segmented regions of the image. The segmented image was subsequently fed into the memory module that squeezed the features of the image. The max-pooling blocks were the major components of the squeeze process, suppressing the inefficient and enhancing the informational features. Batch normalization and bottleneck layers were also deployed to reduce the complexity and improve the stability of the architecture, respectively. The excitation, squeeze, and bottleneck processes within the memory module provided a robust feature extraction and reduced the overall complexity of the architecture. The extraction of the optimal features is fundamental to the accuracy of the CNN performance, with a minimal increase in the number of total parameters. Finally, a ReLu activation was performed on the computed results using an FC followed by a classification layer after SoftMax activation. The central CNN module consisted of a basic convolutional layer architecture, ending with an FC layer.

A region-based fast CNN (RFCNN) [131,132,133] and CNN two-stage method was proposed by [134]. The RFCNN carried out an automatic detection of the lesions and marked the RoIs of those lesions. The CNN used for classification was based on transfer learning and the attention mechanism [135], with the Kaggle and MESSIDOR datasets used for the evaluation of the performance. Two convolutional neural network-based models were also presented in [136]. A DL model, which consisted of CNN512 and YOLOv3 [137], classified images into five classes. First, the CNN512 model classified the input image, and the input image was fed into the YOLOv3 model, which detected and localized the lesions. Lastly, the CNN512 and YOLOv3 models were fused to improve the performance of the final classification. A hybrid model that consisted of two phases, a pre-processing phase and deep learning phase, to improve the classification results was described in [138]. Histogram equalization and contrast limited adaptive histogram equalization algorithms were used to pre-process the images and the CNN for classification.

The authors of [139] detailed an automated knowledge model that detected key antecedents using fundus images in the classification of DR. A convolutional neural network (CNN), back propagation neural network (BPNN), and deep neural network (DNN) were tested, the knowledge model calculating the weights that yield the severity level of the eye. After the weights were calculated, the fuzzy c-means algorithm was used to detect the target class thresholds. The results showed that the proposed model successfully identified the proper class of the severity from the DR images.

In the DCNN and linear support vector machine (LSVM)-based model presented by Burlina et al. [140], the former extracted the features from the fundus image, with the latter performing the classification of the age-related macular degeneration (AMD). Following the resizing of the images into 231 ± 231 pixels, the ImageNet dataset was used for the pre-training of the OverFeat CNN. The NIH AREDS [141] dataset, comprising four categories of AMD severity, was used for the validation of the model. Another two-stage approach [142] firstly extracted the texture features from the image using local binary patterns (LBP), formulated as (Equation (8)):

(8) $$LBP\left(x_{c},y_{c}\right)=\overset{p-1}{\underset{p=0}{\sum }}s\left(g_{p}-g_{c}\right)2^{p}\;$$

where $g_{c}$ represents the gray value of the central pixel and $g_{p}$ is the gray value of the p adjacent pixel to the central pixel. The function s(x) can be denoted as:

$$s\left(x\right)=\{\begin{array}{c} 1,\;\;\;\;\;x\geq 0\\ \;0,\;\;\;\;\;\;otherwise\;\;\;\;\;\;\;\;\; \end{array}\;$$

Subsequently, a number of DL-based algorithms, e.g., DenseNet, ResNet, and DetNet [143], were explored to yield a final classification using the extracted texture features.

A comparison of three CNNs, ResNet50, DenseNet, and VGG16, for the classification of fundus fluorescein angiography (FFA) was presented in [144]. Annotations were formed to locate four different types of lesions, viz., leakages, micro-aneurysms, the non-perfusion region (NP), and laser scars. Furthermore, during the training of these models, the cross-entropy function was used as a loss function, formulated as (Equation (9)):

(9) $$C=-\frac{1}{n}\overset{n}{\underset{i}{\sum }}\left[y_{i}ln\left(a_{i}\right)+\left(1-y_{i}\right)ln\left(1-a_{i}\right)\right]\;$$

where *C* is the cross-entropy cost function, $y_{i}$ represents the output for the ith neuron, $a_{i}$ is the actual output, n donates the number of neurons involved in the calculation, and I is the total number of neurons. The stochastic gradient descent (SGD) algorithm was used as an optimizer for all networks. The comparison of all the considered models indicated that the DenseNet achieved the most effective results.

#### 2.4.2. Deep Belief Neural Networks

The nonlinear environments and dimensions of the features affect the diagnosis and classification process. A deep belief neural network (DBNN) and SVM-based framework was proposed for the extraction of the features [145], also reducing the dimensions of the features for the classification process. The DBNN received the retinal images as an input and extracted the deep features. The generalized regression neural network (GRNN) [146] was used subsequently for the reduction and selection of the optimal features. The extracted features were fed into an SVM for the classification.

#### 2.4.3. Deep Neural Networks (DNNs)

A range of DL approaches used hand-crafted features for the classification and grading of the DR severity. A quadrant ensemble framework which consisted of deep neural network and Inception-ResNet-V2 was proposed in [147] for the automatic grading. The framework also incorporated optical disc localization, histogram equalization, quadrant cropping, and data augmentation to improve the network performance. The MESSIDOR dataset was used for the training and the latest IDRiD dataset was used for the validation of the framework.

#### 2.4.4. Other Variants of Deep Learning (DL) Models

A study that aimed to reduce the dimensionality during the data pre-processing is presented in [148]. PCA extracted the most important features from the dataset and the firefly algorithm was then used to reduce the dimensionality. The reduced dataset was fed into the DNN for classification.

A number of DR factors that included the retinal condition of the eyes, diameter of the optical disc (OD), presence of the micro-aneurysms (MAs), and the Euclidian distance between the center of the OD and macula were extracted systematically using the technique described in [149]. A fuzzy analytical network was applied to rank the important attributes of the DR form, ranked from the most to the least important. The transformed fuzzy neural network was created to enhance the classification accuracy. The association rules extracted from the selected DR attributes were used to determine the degree of severity. The approach targeted the early identification and study classification of DR in advance of the onset of criticality, improving the quality of patient care.

A U-Net model was used [150] for the segmentation and classification of the retinal vessels. A range of settings of batch normalization and dropout for the U-Net model were evaluated to investigate the effect of retina vessels in DR classification. The pre-trained Inception-V1 network was used for the classification of the DR severity. The MESSIDOR dataset was used to create the two datasets of the retinal images, without and with the presence of the vessels. The results confirmed that the retinal vessel is one of the optimal features for the classification of DR throughout the range of stages of severity, from early to severe.

A data-driven deep learning method that used colored fundus images proposed in [151] classified multiple classes on the basis of the level or the stage of the eye infection. A set of 170 colored fundus images acquired from diabetic patients were used to train and test the model. The pre-processing phase of the two-stage model resized the images according to the input layer size of the network. A channel size of 3 and an image size of 70–100 were set for the RGB image. Noise was removed from the images by a Gaussian filter (GF) and a multi-support vector machine (MSVM) extracted the non-critical and critical features from the images, assigning them an appropriate classification.

An automatic image level DR classification system that consisted of multiple DL models was reported in [152]. The Ada boost algorithm was used for the integration of the different DL models, reducing the bias of any individual model. A weighted class action maps (CAMs) method was applied to highlight the suspected position of each lesion. The interpretable ensemble DL model was shown to be more robust and to yield superior performance compared to an individual DL model.

A deep multi-task DR grading (DeepMT-DR) model with the capability to operate using low-resolution (LR) images, which simultaneously executed the auxiliary tasks of the image super resolution (ISR) and lesion segmentation, was detailed in [153]. The model consisted of three hierarchical layers; the first processed the ISR images, the second segmented the lesions, and the third performed the grading. Moreover, a loss-aware task was deployed in the second layer that encouraged the ISR to focus on the pathological regions, which improved the classification accuracy. The performance of the supervised DL models was compromised by the limited availability of high-quality adjudicated labels during their training phase. An automated sans-coding approach based on a teacher–student model was proposed to address this issue [154]. A teacher model was developed from a small, high-quality labeled dataset. Subsequently, Google Cloud automated machine learning (Auto-ML) was used to the train the image classification sans-coding, which assigned high-quality adjudicated DR severity labels to the color fundus images. The teacher model also generated DR referral predictions for the unlabeled fundus images. The teacher model, trained using high-quality images, was combined with image–label pairs for the training of the student model. The approach used self-training to decrease the over-fitting and increase the classification performance. In addition, high-quality severity labels were generated, which support clinical experts to carry out a diagnosis.

Different combinations of datasets and convolution networks were evaluated in the development of a stochastic coordinate descent deep learning (SCDDL) architecture reported in [155]. The selected models are implemented, through a layer-by-layer comparison of the convolution matrix, transition, the pooling layer and dense layer of each network according to their matrix order. Loss minimization, considered as an objective function after the prediction, was carried out at every stage of the networks. The generalized function of the loss minimization for each network was formulated as (Equation (10)):

(10) O(x)=LxϵNmin(x) 

with the loss function for each convolution network derived as follows:

(11) (xL)xϵCNNL=(xN)xϵCNNO−O(x)    

The main objective of this framework is to identify the layers which are central to the optimization of the matrix order, with the minimization performed for the classification of the DR severity levels.

The automatic synergic deep learning (SDL) model introduced in [156] consisted of three stages: pre-processing, segmentation, and classification. The redundant noise at the edges of the images was removed at the pre-processing stage. The region of interest was then extracted through a histogram-based segmentation. Lastly, the segmented image was fed into an SDL for classification. The SDL comprised three major components, an input layer, k DCNNs, and $C_{K}^{2}$ synergic networks (SNs). Every DCNN element generated an independent learning depiction of the input data. The SNs included a model of a fully connected structure that ensured the input layer consistently operates on similar classes and provides the remedial corrections of the synergic errors.

The first stage of a hyper-parameter-tuning Inception-V4 (HPTI-V4) model proposed in [157] was pre-processing, where the contrast limited adaptive histogram equalization (CLAHE) function was applied to enhance the contrast level of the images. The region of interest (RoI) was then segmented through a histogram-based segmentation, and the segmented image was subsequently fed into the HPTI-v4 for the extraction of the lesion features. The Bayesian optimization (BO) function, which selected the subsequent parameters in an informed manner, was applied for the tuning of the HPTI-v4. Finally, a feed-forward artificial neural network (ANN) was used for the classification, with the MESSIDOR dataset selected for the validation of the model.

The DR|GRADUATE system, which is able to deal with the ordinal nature of DR grading, was proposed in [158]. A novel Gaussian sampling approach based on multiple instance learning was used to support the system and to learn the explanation maps and the prediction uncertainty for the associated grades during training in the image-wise label phase. The system must predict the generalized Bernoulli distribution biased around the classes for each image. The Gaussian distribution added the bias into the model to compute the image-wise grade probability.

A multi-task hierarchical neural network-based framework that related the severity levels and relevant features simultaneously was introduced in [159]. The architecture featured two heads and one backbone, the former being two independent forward neural networks, one for the feature selection, the other for the grading of the severity levels. The squeeze-and-excitation (SE) network [43] was used for the extraction of the features at higher scales at the backbone. The features extracted by the relevant head were used as inputs, together with the skip-connection to the severity head, to assist in the detection.

A ResNet and gradient-weighted class activation mapping (Grad-CAM)-based multi-label model that automatically located the relevant lesions and reduced the annotation burden was reported in [160]. The assigned labels of the located lesions for all fundus images were used for classification.

#### 2.4.5. Deep Learning in a Clinical Environment

Although, in recent years, state-of-the-art deep learning models have been evaluated during the development process, the deployment and performance validation of DL-based systems within clinical environments remains an open research challenge [161]. However, a number of deep learning-based interactive techniques aimed at increasing the trust of the patients have been assessed in controlled laboratory settings by pathologists [162]. Thus, the use of deep learning for classifications within a clinical environment is summarized in the following section.

A patient-centric deep learning system was deployed in a real-world clinical setting in a study, reported in [163], to assess its role in, and determine its benefits for, the screening workflow, assess user expectations from a DL system, and to garner post-deployment experiences. The results indicated that different socio-environmental factors affected the performance of the model, the patient experience, and nursing workflows. The authors of [164] detailed the results of a comparison of the performance of a DL system deployed on a large clinical scale using human graders. A total of 25,326 gradable retinal images of patients were collected through a community-based screening program across Thailand, and these images were used for the validation of the system, with international retinal specialists assigning the grades. A direct comparison of the outputs of the DL system with the actual grades assigned by pathologists for the same population indicated that the automatic DL system performed nearly as well as the human graders. The quadratic-weighted kappa values used for the evaluation of the DR severity levels by the system and the human graders were 0.85 and 0.78, respectively. A summary and details of the performance of the reported methods are given in Table 4.

### 2.5. Validation of Deep Learning Models for DR Analysis

A CNN-based learning system for the detection and validation of DR proposed in [127] had the ability to identify glaucoma, referable diabetic retinopathy, and age-related macular degeneration (AMD) from retinal images. The CNN architecture differentiated between these conditions through the gradual optimization of the weight parameters of the model. The combination of the VGG16, spatial pyramid pooling layer (SPP) [165], and network-in-network (NiN) [166]—referred to as the VGG-NiN model—proposed in [124] extracted the highly nonlinear features from the color fundus images and executed processing at any scale, owing to the SPP layers. The model achieved an acceptable detection accuracy, as the stacking of the NiN has been shown to treat significant degrees of non-linearity.

A deep learning algorithm (DLA) developed for the validation of the DR [167] used, as a baseline, a 3 × 640 × 640 input retinal image size obtained through a pre-processing step that trimmed the external background borders of the image. The model was based on a convolutional network and consisted of 17 layers and 391,325 parameters. The layers of the model were characterized as feature extractors and classifiers. Each layer comprised a stack of 3 × 3 convolutional layers, with a 1 × 1 stride and 1 × 1 padding, followed by batch normalization and a ReLu activation function. A set of 38,694 different retino-graphic images were used for the training and validation of the DLA. Firstly, the DLA read the images and, then the reading was performed by four masked senior retina ophthalmologists. The DLA supported the diagnosis by identifying when the fundus image had at least four micro-aneurysms, along with or without soft or hard exudates in the absence of the other known causes of the changes. The model also further classified the images with respect to the severity levels. A summary of the performance of the reported methods is given in Table 5.

## 3. Overview

An extensive body of research has been reviewed, the conclusions of which indicate that deep learning techniques, algorithms, and methods have the potential to yield systems of value executing the segmentation, prediction, and classification, and forming the basis for decision support applications that enhance the execution of key tasks within DR diagnosis. A large diversity of DL models, architectures, applications, methods, and frameworks have been considered. Pre-trained CNNs are preferred as a feature extraction method. Deep convolutional neural network (DCNN), deep neural network (DNN), generative adversarial network (GAN), and U-Net have all been used effectively to support the analysis of DR. With respect to transfer learning, a number of pre-trained networks are currently accessible on different public repositories that can be downloaded and applied to the treatment of any retinal image format. Although existing systems and frameworks are predominately founded on hand-crafted features produced by pathologists, the use of end-to-end trained CNN models for the analysis of medical images (like retinal images) are beginning to enjoy increased adoption. Furthermore, DL rather than ML trained on traditional hand-crafted features has become the preferred methodology for generating models that are beginning to be integrated into the existing retinal image analysis tools.

## 4. Key Factors in Successful Deep Learning Methods

Given the wide-ranging review of a significant number of reported techniques related to DR analysis, the expectation would be to provide clear guidance on the design of the optimum DL models, architecture, framework, or approach as functions of a key individual task or application. However, although CNN-based methods have yielded better results compared to other deep learning algorithms, a striking conclusion is that the exact architecture of any DL model is not a critical determinant in creating an effective solution. A number of reported developments have used the same network architecture, but extensive variations in performance are evident [138,139]. Implementing variations in the number of layers of CNN networks to improve performance is a well-known approach in the domain of expert knowledge. The pre-processing stages and data augmentation are also key to the development of high-performing deep learning models. A range of normalization techniques have been explored as pre-processing steps to improve the generalization of the networks without significant changes to their core architectures. The application of data augmentation strategies and pre-processing techniques improves the overall robustness and performance of the models, playing a very important role in yielding effective solutions for DR analysis through deep learning. Furthermore, task-oriented network architectures, such as multi-scale and multi-view architectures, achieve better performance in comparison with traditional CNNs.

**Figure 4:**
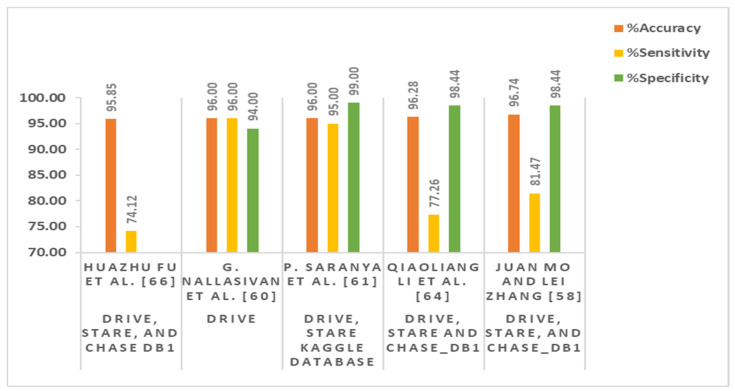
Top 5 publications reporting the best performance for the segmentation.

**Figure 5:**
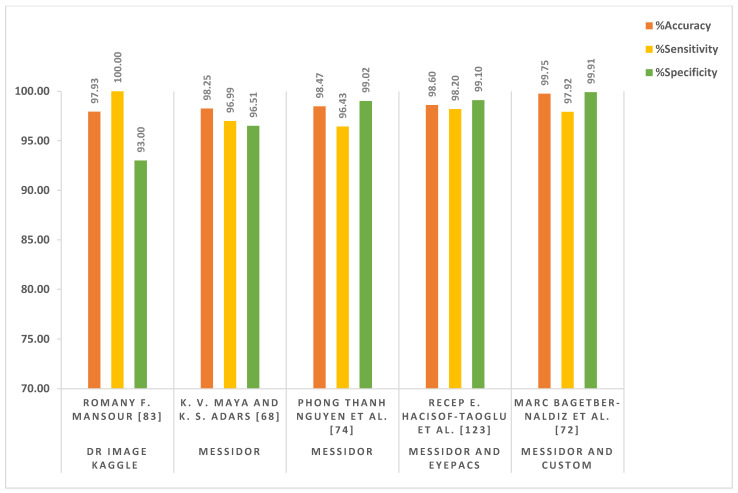
Top 5 publications reporting the best performance for the prediction.

**Figure 6:**
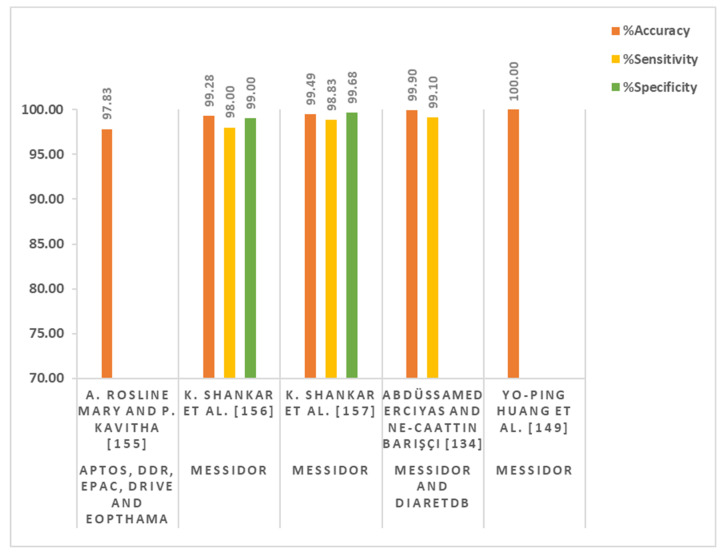
Top 5 publications reporting the best performance for the classification.

Model designs must be driven by the receptive field and input image size, i.e., the single output correspondence with respect to the spatial area of the input. The selected input size must meet the criteria for the resolution and the context, governed by the requirements of the application. For example, although, on occasion, a variation in the receptive fields of the network may produce effective results, changes or increments in the patch size in the search for an enhanced context has not been beneficial in all cases. Evaluations of the impact of the visual input of the network for a task indicate that, in the case of high inputs, a modification to the network architecture was necessary in order to achieve the effective results. The optimization of the hyper-parameters, e.g., the learning rate and dropout rate, impacts the overall performance of the network, and it is surprising that more research on the methods or techniques required to optimize the best set of hyper-parameters for an application has not been carried out. There is an evident trend in the implementation of transfer learning methodologies, exemplified by the growing use of pre-trained networks to create solutions that support the analysis of DR. The ResNet-based network has been harnessed extensively in this respect to achieve effective results. The validation of the functionality, performance in operational environments, and value of deep learning-based decision support in clinical settings is only just beginning. However, more extensive deployments are required to acquire sufficient data that sheds light on the optimization of the performance and, in so doing, reduces the barriers to adoption. The five publications reporting the approaches that provide the best performance for each key task-segmentation, prediction, and classification are shown in Figure 4, Figure 5 and Figure 6, respectively.

## 5. Limitations, Research Gaps and Future Directions

Despite the scope of the reported research, this review of the advances in the discipline has nevertheless identified limitations in the current approaches, highlighted the gaps in the research that have surfaced to date, and signposted the future directions for the development of deep learning solutions that support the analysis of DR. A proliferation in the number of reported models and methods based on DL is evident in the recent past, and most, if not all, of these DL-based models are presented as ‘black boxes’, i.e., the solutions do not provide interpretations of their diagnostic value, which hinders their widespread use in operational clinical environments. Advances in interpretable DL techniques must be pursued in the future to overcome this barrier to their use.

The effective training of deep learning models requires appropriately sized datasets, their ready availability in the applications under review remaining an open gap, especially with respect to segmentation and validation. The recent migration towards the use of high-definition cameras to capture retinal images of the eye is beginning to generate an increasing number of appropriate images. In similar fields, such as medical imaging, several PAC systems have been installed in a number of healthcare centers and hospitals, but the use of these systems remains limited in the domains of ophthalmology and pathology. Furthermore, well-structured digital archives offer a limited number of retinal images. In many studies, out of necessity, custom datasets with large numbers of images have been created, but they are not rendered openly accessible to the extended research community. Moreover, the retinal fundus images currently available are characterized by a lack of uniformity, e.g., the images were captured under different conditions. They are also characterized by variations in the illumination, e.g., the non-uniform diffusion of light in the retina, sphere shape of the retina, the same angle not having been used for all captured images. Another limitation is out-of-focus images, e.g., the use of different cameras and resolutions for all the captured images. A major gap to be addressed, therefore, is the ready access to an appropriate scope and number of retinal images stored in open repositories to act as a foundation that accelerates the development/training/optimization of the performance of existing and new deep learning models. Consideration of these issues should be at the forefront of strategies defining the experimental protocols for the acquisition of custom or new datasets.

The visualization and understanding of the features used by deep learning algorithms for the optimization of learning and forming of accurate predictions also remain gaps in the research to date. The combination of traditional and DL-based systems for the selection of the most appropriate features and detection of health conditions is a worthy and challenging area of research. DL-based DR feature maps combined with traditional features could potentially improve the accuracy of the predictions and, in turn, provide the robust validation of the value of DL systems demanded by clinicians. Furthermore, the use of deep learning and local descriptors for the pathological features are at the core of effective DR analysis. All developments of deep learning models invariably benefit from consultation by subject matter experts who can provide a more precise understanding of the important pathological features. The detection and classification of other eye diseases, such as AMD and glaucoma, from color fundus images can potentially bring benefits to the optimization of the screening process. The combination of patient data, such as medical history and demographics, with the prediction will enhance the precision of the decision regarding whether or not to refer individual DR patients.

The introduction of text-mining methods and techniques within the development of deep learning models will create benefits by facilitating reporting on annotations and fostering the ability to change the structure of the labels in an automatic manner. The DR analysis community has an expectation that generating reports for label structuring will become ever more burdensome. The use of the structured and text-free report would also bring value in enhancing the training of the networks for DR analysis. It is need to encourage domain experts, e.g., ophthalmologists, to allocate time to generating task-specific reports, e.g., screening, segmentation, prediction, classification, and validation reports, and text-free reports from retinal image data will be of undoubted value in the training of DL algorithms. The labeling of the retinal images is a time-consuming process, requiring a high level of expertise for its execution. Thus, the extraction and labeling of features is a rewarding area of research, as solutions would improve the overall performance of the models used for DR analysis. The availability of algorithms performing efficient slice-by-slice annotations is limited, and there remains scope for innovation.

EyePACS and MESSIDOR-2 datasets have been used most commonly for the training and validation of algorithms. The training and validation of the DL models and systems through these data sets, however, requires consideration regarding the impacts of noise and uncertainty and the approaches to these impacts. Although a few studies have attempted to determine the impacts of label uncertainties directly through the use of an appropriate loss function, the scope for research in this domain is significant. Class imbalance is also a key issue related to the data and, although several data augmentation techniques are available, which can be used to generate the new retinal images with blood vessels and lesions by rotating and scaling the images, additional validation is needed to confirm that these approaches do not increase the imbalance.

Deep learning models and architectures in DR analysis still face the issue of patch classification, as the anatomical location of the patch is not known. A potential solution to this issue is to feed the entire retinal image into the deep network so as to achieve its learning capabilities. Only one approach was founded on the introduction of a loss function, such as which is based on the dice coefficient method [151]. However, the feeding of the entire image into a network may not be feasible in every case due to some constraints placed by restricted access to GPU resources, limited memory size, and bandwidth, as retinal images fall in the range of giga pixels. Furthermore, a network characterized by small receptive fields is not able to operate on an entire image. Thus, the definition and design of the methods and techniques able to operate on entire images is a fruitful area of research.

Another outstanding research challenge is that, generally, most CNN-based networks and models use a kernel of a fixed size for the slicing of images, which results in the loss of important information hidden in unexplored regions. Thus, the exploration of the impact of a variable kernel size, instead of a fixed size, on model performance for the slicing of the image data would increase our understanding of the optimum designs. Moreover, most deep learning models reported to date have been developed assuming that their input is a retinal image. However, in some operational environments, a tempered and not a real retinal image may be the only input available. Therefore, the development of user-friendly image editing software is required to temper an image. Equally important in this respect is the deployment of intelligent computer-aided systems that are able to confirm that the input image is indeed an authentic retinal image before further processing.

Finally, studies have provided evidence that, although deep learning models yield effective results in experimental, laboratory-level conditions, a loss of performance is incurred when they are evaluated in a clinical environment. A limited number of studies on the challenges and impacts of implementing DL models in clinical settings have been reported. Thus, research opportunities exist in regard to our gaining of an understanding of the routes to the implementation of DL models in clinical settings and the validation of the performance during a real-time clinical examination for the major tasks of segmentation, prediction, and classification, which are all core to effective decision support in DR analysis. In order to accelerate trust in, and the adoption of, DL-based intelligent systems, they must be developed in consultation with expert ophthalmologists and validated in operational clinical settings, e.g., in environments where retinal images are captured under different conditions, such as poor focus/contrast, poor pupil dilation, cataracts, on patient samples of differing ethnicities, and qualities of systemic control (good and poor control).

## Figures and Tables

**Table 1:** A comparison of reported deep learning techniques for screening and recognition.

Study	Proposed Solution	Languages/Libraries Software/Tools for Simulation Environment and Implementation	Data Set	Number of Images Used	Image Modalities	Evaluation of Performance
Yi-Ting Hsieh et al. [35]	CNN and Inception-V4 network-based software named VariSeeTM	Not Mentioned	Custom-developed at National Taiwan University Hospital between July 2007 and June 2017 + EyePACS	39,136	Color fundus images	Maximum accuracy = 98.4%
Silvia Rego et al. [36]	CNN model with Inception-V3 based software	Not Mentioned	EyePACS	350	Color fundus images	Sensitivity = 80.8% Specificity = 95.6% PPV = 77.6% NPV = 96.3%
Fethallah Benmansour et al. [37]	Inception-V3 model with transfer learning based automatic screening approach	Not Mentioned	Custom-developed at Inoveon Corporation, Oklahoma City, UK	1,790,712	7-field color fundus photographs (7F-CFP)	Area under the receiver operating characteristic (AUROC) curve = 96.2% Sensitivity = 94.2% Specificity = 94.6%
Sajib Kumar Saha et al. [38]	Deep convolutional neural network-based approach	Not Mentioned	EyePACS	7000	color fundus images	Accuracy = 100% Sensitivity = 100% Specificity = 100%
Tao Li et al. [39]	DL framework consisting of VGG-16, ResNet-18, GoogleNet, DenseNet-121, and SE-BN-Inception	Nvidia Tesla K40C GPU	Custom-developed, collected from 147 hospitals from 2016 to 2018, covering 23 provinces in China, 84 of which are grade-A tertiary hospitals.	13,673	color fundus images	Maximum accuracy = 95.74%
Qaisar Abbas et al. [45]	Gradient location orientation histogram (GLOH), DColor-SIFT, deep learning neural network (DLNN), restricted Boltzmann machines (RBMs), and Shannon entropy constraints (SECs)-based system	MATLAB R2015a, Core i7 64-bit Intel processor system with 8 GB DDR3 RAM	DIARETDB1, MESSIDOR, and custom-developed at Private Hospital Universitario Puerta del Mar (HUPM, Cádiz, Spain)	750	color fundus images	Sensitivity = 92.18% Specificity = 94.50% Area under the receiving operating curves (AUC) = 92.4%

**Table 2:** A summary of the reported techniques for retinal blood vessel segmentation.

Study	Proposed Solution	Languages/Libraries Software’s/Tools for Simulation Environment and Implementation	Data Set	Number of Images Used	Image Modalities	Evaluation
Kevis-Kokitsi Maninis et al. [53]	Deep convolutional neural networks (DCNNs) and VGG net-based model	NVIDIA TITAN-X GPU is 85 milliseconds (ms)	DRIVE and STARE	60	Color fundus images	Maximum precision = 83.1%
Aaron Wu et al. [54]	Deep convolutional neural network (CNN) and principal component analysis (PCA)-based framework	Not mentioned	DRIVE	20	Color fundus images	AUC = 97.01%
Jen Hong Tan et al. [55]	Seven-layer CNN model	MATLAB, Intel Xeon 2.20 GHz (E5-2650 v4) processor and a 512 GB RAM	DRIVE	40	Color fundus images	Accuracy = 94.54%
Huazhu Fu et al. [57]	Fully connected conditional random field (FCCRF) and FCN-based method	Caffe library, NVIDIA K20 GPU	DRIVE and STARE	60	Color fundus images	Maximum accuracy = 95.45% Sensitivity =71.40%
Juan Mo and Lei Zhang [58]	Multi-level hierarchical features-based fully convolutional network (FCN) model	NVIDIA GTX Titan GPU	DRIVE, STARE, and CHASE_DB1	88	Color fundus images	Maximum accuracy = 96.74% Sensitivity = 81.47% Specificity = 98.44% AUC = 98.85% Kappa = 81.63%
G. Nallasivan et al. [60]	Principle component analysis (PCA), gray level co-occurrence matrix (GLCM), and CNN-based technique	Not mentioned	DRIVE	40	Color fundus images	Accuracy = 96% Sensitivity = 96% Specificity = 94%
P. Saranya et al. [61]	CNN and VGG-16 net-based architecture	Python, Keras version 2.3 and TensorFlow version 1.14, Intel(R) Core (TM) i7-6700HQ Cpu@2.60 gHz, 16 GB RAM, Nvidia GeForce GTX 960 GPU	DRIVE, STARE, Kaggle database	2260	Color fundus images	Maximum accuracy = 96% Specificity = 99% Sensitivity = 95% Precision = 99% F1 score = 97%
Debapriya Maji et al. [62]	Deep neural network (DNN) and stacked ne-noising auto-encoder-based hybrid architecture	Not mentioned	DRIVE	40	Color fundus images	Maximum average accuracy = 93.27% Area under ROC curve = 91.95%, Kappa = 62.87%
Abhijit Guha Roy and Debdoot Sheet [63]	Stacked auto-encoder (SAE)-based deep neural network (DNN) model	Not mentioned	DRIVE	40	Color fundus images	Area under ROC curve = 92%
Qiaoliang Li et al. [64]	Deep neural network (DNN) and de-noising auto-encoders (DAEs)-based supervised approach	MATLAB 2014a, AMD Athlon II X4 645CPU running at 3.10 GHz with 4 GB of RAM	DRIVE, STARE, and CHASE_DB1	88	Color fundus images	Maximum accuracy = 96.28% Sensitivity = 77.26%, specificity = 98.44% AUC = 98.79%
Avisek Lahiri et al. [65]	Stacked de-noising auto-encoders (SDAEs) and convex weight average (CWA)-based two-level ensemble approach	Not mentioned	DRIVE	40	Color fundus images	Maximum average accuracy = 95.33% Kappa = 70.8%
Huazhu Fu et al. [66]	Conditional random field (CRF) and CNN as a recurrent neural network (RNN)-based method	Caffe library, NVIDIA K40 GPU	DRIVE, STARE, and CHASE DB1	88	Color fundus images	Maximum average accuracy = 95.85% Sensitivity = 74.12%

**Table 3:** Summary of reported techniques for lesion detection.

Study	Proposed Solution	Languages/Libraries Software/Tools for Simulation Environment and Implementation	Data Set	Number of Images Used	Image Modalities	Evaluation
Filippo Arcadu et al. [8]	Deep convolutional neural network (DCNN), Inception-V3 network, and Random forests (RFs)-based model	Keras using Tensor Flow	Custom-developed in the RIDE (NCT00473382)33–35 and RISE (NCT00473330)	14,070	7-field color fundus Photographs (CFPs)	Maximum area under the curve (AUC) = 79% Sensitivity = 91% Specificity = 65%
Carson Lam et al. [67]	CNN and weight matrix-based method	Tesla K80 GPU hardware device, Tensor Flow, and OpenCV	MESSIDOR-1	36,200	Color fundus images	Maximum accuracy = 74.1% Sensitivity = 95%
K. V. Maya and K. S. Adars [68]	Recursive region growing segmentation (RRGS), Laplacian–Gaussian filter (LGF), and CNN-based model	Not mentioned	MESSIDOR	1200	Color fundus images	Accuracy = 98.25% Sensitivity = 96.99% Specificity = 96.51%
C. Rajaa and L. Balaji [69]	Adaptive histogram equalization (AHE) + fuzzy c-means clustering (FCM) and CNN-based model	Intel (R) Core i5 processor, 3.20 GHz, 4 GB RAM, Microsoft Windows 7, and Matlab	Diabetic retinopathy database	76	Color fundus images	Accuracy = 93.2% Specificity = 99% Sensitivity = 98.1%
Shikhar Seth and Basant Agarwal [70]	CNN and Linear support vector machine (LSVM)-based model	Not mentioned	EyePACS	35,126	Color fundus images	Sensitivity = 93% Specificity = 85%
Akhilesh Kumar Gangwar and Vadlamani Ravi [71]	Inception-ResNet-v2 and CNN-based model	Keras framework	MESSIDOR and APTOS 2019	4862	Color fundus images	Accuracy = 82.18%
Marc BagetBernaldiz et al. [72]	CNN-based method	Not mentioned	Custom developed at healthcare area (University Hospital Saint Joan, Tarragona, Spain) and MESSIDOR	16,186	Color fundus images	Maximum accuracy = 99.75% Sensitivity = 97.92% Specificity = 99.91% Positive Predictive Value (PPV) = 98.92% Negative Predictive Value (NPV) = 99.82%
Tang F et al. [73]	CNN (ResNet-50) and transfer learning-based system	Not mentioned	Custom-developed	9392	Ultra-Wide Field Scanning Laser OphthalMoscope (UWF-SLO)	Area under the receiver operating characteristic curve (AUROC) = 92.30% Sensitivity = 86.5% Specificity = 82.1%
Phong Thanh Nguyen et al. [74]	An ensemble of orthogonal learning particle swarm optimization (OLPSO)-based CNN model (OLPSO-CNN)	Python 3.6.5	MESSIDOR	1200	Color fundus images	Accuracy = 98.47% Sensitivity = 96.43% Specificity = 99.02%
P Saranya and K M Umamaheswar [76]	CNN-based framework	Not mentioned	MESSIDOR	1200	Color fundus images	Accuracy = 97.54% Sensitivity = 90.34% Specificity = 98.24%
Nagaraj G et al. [77]	CNN and VGG-16 network-based framework	Not mentioned	EyePACS	35,126	Color fundus images	Maximum accuracy = 73.72%
Stuart Keel et al. [78]	CNN independent adaptive kernel visualization technique	Not mentioned	The images were collected from different hospitals in China between March 2017 and June 2017	100	Color fundus images	True positive ratio (TPR) = 96% False positive ratio (FPR) = 85%
Gen Min Lin et al. [79]	CNN-based architecture for entropy images	Matlab	EyePACS	33,000	Color fundus image and entropy images	Accuracy = 86.10% Sensitivity = 73.24% Specificity = 93.81%
Lei Zhou et al. [81]	CNN-based multiple instance learning (MIL) technique	4 NVIDIA GeForce GTX TITAN X GPUs	Diabetic retinopathy detection dataset on Kaggle, MESSIDOR, and DIARETDB1	36,415	Color fundus images	F1-score = 92.4% Sensitivity = 99.5% Precision = 86.3%
Cam-Hao Hua et al. [82]	Skip-connection deep networks (Tri-SDN) architecture	Pytorch, Scikit-learn and NVIDIA 1080TI GPU	Custom-developed at Kyung Hee University Medical Center, Seoul, South Korea	96	Color fundus images	Accuracy = 90.6% Sensitivity = 96.5% Precision = 88.7% Specificity = 82.1% Area Under Receiver Operating Characteristics = 88.8%
Romany F. Mansour [83]	AlexNet DNN-based computer-aided diagnosis (CAD) system	MATLAB 2015a	DR image Kaggle	35,126	Color fundus images	Accuracy = 97.93% Sensitivity = 100% Specificity = 93%
Yukun Guo et al. [84]	U-Net and CNN-based assessment model	Not mentioned	Custom-developed at Oregon Health and Science University	1092	Montaged wide-field OCT angiography (OCTA)	
Gaurav Saxena et al. [86]	Inception-V3 and ResNet-V2-based hybrid model	2 x Intel Xeon Gold 6142 processor, 2.6 GHz, 22 MB cache, 384 GB memory	EyePACS and MESSIDOR-1	58,039	Color fundus images	Maximum accuracy = 95.8% Sensitivity = 88.84% Specificity = 89.92%
Cristina Gonzalez-Gonzalo et al. [87]	CNN-based Ret CAD v1.3.0 system	Not mentioned	DR-AMD and age-related eye disease study (AREDS)	8871	Color fundus images	Maximum AUC = 97.5% Sensitivity = 92% Specificity = 92.1%
Varun Gulshan et al. [88]	Deep convolutional neural network (DCNN)-based model	Not mentioned	EyePACS MESSIDOR	128,175	Color fundus images	AUC on EyePACS = 99.1% Sensitivity on EyePACS = 97.5% Specificity on EyePACS = 93.4% AUC on MESSIDOR = 99% Sensitivity on MESSIDOR = 96.1% Specificity on MESSIDOR = 93.9%
SEHRISH QUMMAR et al. [92]	Ensemble approach which consists of five different deep CNN models that include Inception-V3, Resnet50, Dense-121, Dense-169, and X-ception	NVIDIA Tesla k40 containing 2880 CUDA, core CuDNN, Keras, Tensor Flow	Kaggle	35,126	Color fundus images	Maximum accuracy = 80.8% Recall = 54.5% Specificity = 86.7% Precision = 63.8% F1-Score = 53.7%
Kangrok Oh et al. [93]	Residual network with 34-layer (ResNet-34)-based model	Not mentioned	Custom-developed at Catholic Kwandong University International St. Mary’s Hospital, South Korea	11,734	Ultra-wide-field fundus Images	Accuracy = 83.38% AUC = 91.50% Sensitivity = 83.38% Specificity = 83.41%
Ling Dai et al. [96]	ResNet and Mask-RCNN-based deep DR system	x86 compatible CPU, 10 GB free disk space, at least 8 GB main memory, Python version 3.7.1	Custom-developed at Shanghai Integrated Diabetes Prevention and Care Center	666,383	Color fundus images	AUC = 94.2 % Sensitivity = 90.5% Specificity = 79.5%
Gazala Mushtaq and Farheen Siddiqui [98]	Densely connected convolutional network (DenseNet-169)-based system	OpenCV, Tensor Flow and Scikit-learn	Aptos 2019 blindness detection and diabetic retinopathy detection	7000	Color fundus images	Accuracy = 90.34% Cohen kappa score = 80.40%
Yashal Shakti Kanungo et al. [99]	Inception-V3-based architecture	Python, OpenCV	EyePACS	40,000	Color fundus images	Accuracy = 88% Specificity = 87% Sensitivity = 97%
Toshihiko Nagasawa et al. [100]	Ultra-wide-field fundus image-based deep convolution neural network (DCNN) model	Python, Keras, Tensor Flow	Custom-developed at the ophthalmology departments of Saneikai Tsukazaki Hospital and Tokushima University Hospital from April 1, 2011, to March 30, 2018	378	Ultra-wide-field fundus images	Sensitivity = 94.7% Specificity = 97.2% AUC = 96.9%
Jaakko Sahlsten et al. [103]	DCNN and Inception-V3 network-based deep learning system (DLS)	Not mentioned	Custom-developed at Digifundus Ltd., which provides diabetic retinopathy screening and monitoring services in Finland	41,122	Color fundus images	AUC = 98.7% Sensitivity = 89.6% Specificity = 97.4%
Rishab Gargeya and Theodore Leng [104]	DCNN and deep residual learning (DRL)-based data-driven deep learning algorithm	Intel dual-core processor running at 2.4 GHz	MESSIDOR 2, EyePACS and E-Ophtha databases	75,135	Color fundus images	AUC = 97.0% Sensitivity = 94% Specificity = 98%
Noushin Eftekhari et al. [105]	DCNN and band-pass filters (BPF)-based system	Keras libraries based on Linux Mint operating system with 32 G RAM, Intel (R) Core (TM) i7-6700 K CPU, and NVIDIA GeForce GTX 1070 graphics card	Retinopathy Online Challenge and E-Ophtha-MA	248	Color fundus images	Sensitivity = 77.1%
Ambaji S. Jadhav et al. [107]	Modified gear and steering-based rider optimization algorithm (MGS-ROA) and deep belief network-based model	MATLAB 2018a	DIARETDB1	89	Color fundus images	Accuracy = 93.18% Sensitivity = 86.36% Specificity = 95.45%
Feng Li et al. [108]	Inception-V3 network-based deep transfer learning approach	Intel Core i7-2700 K 4.6-GHz CPU (Intel Corp., Santa Clara, CA), NVIDIA GTX 1080 8-Gb GPU (Santa Clara, CA), Dual AMD Filepro 512-GB PCIe-based flash storage (AMD Corp, Sunnyvale, CA), and 32-GB RAM	MESSIDOR-2	19,233	Color fundus images	Accuracy = 93.49% Sensitivity = 96.93% Specificity = 93.45%
Kh Tohidul Islam et al. [110]	Deep transfer learning (DTL)-based framework which consists of ResNet-18, VGGNet16, Google-Net, AlexNet, ResNet-50, DenseNet-201, InceptionV3, Squeeze-Net, VGGNet-19, ResNet-101, and Inception-ResNet-v2	MATLAB, Intel Xeon Silver 4108 CPU Processor (11 M Cache, 1.80 GHz), NVIDIA QuADro P2000 (5 GB Video Memory), RAM 16 GB, and Microsoft Windows 10	OCT image database	109,309	Ultrasonography, and optical coherence tomography (OCT)	Effective results achieved by DenseNet-201 Accuracy = 97% Specificity = 99% Precision = 97%
Ashish Bora et al. [114]	Inception-V3 network-based system	Not mentioned	EyePACS and custom-developed at National Diabetic Patients Registry in Thailand	575,431	Color fundus images	AUC = 79%
Hidenori Takahashi et al. [115]	Deep neural network-based Google-Net	TITAN X with 12 GB memory	Medical University between May 2011 and June 2015	9939	Color fundus images	Kappa = 74% Accuracy = 81%
Xuechen Li et al. [117]	DenseNet blocks and squeeze-and-excitation block-based optical coherence tomography (OCT) deep network (OCTD_Net)	Keras toolbox, and trained with a mini-batch size of 32, using four GPUs (GeForce GTX TITAN X, 12 GB RAM)	Custom-developed at Wenzhou Medical University (WMU) using a custom-built spectral domain OCT (SD-OCT) system	4168	Optical coherence tomography (OCT)	Accuracy = 92.0% Sensitivity = 90% Specificity = 95%
Varun Gulshan et al. [121]	Deep neural network (DNN)-based algorithm	Python	Custom-developed at Aravind Eye Hospital and Sankara Nethralay between May 2016 and April 2017	103,634	Color fundus images	Sensitivity = 92.1% Specificity = 95.2% Area Under the Curve (AUC) = 98%
Igi Ardiyanto et al. [122]	ResNet-20-based low-cost embedded system	Linux PC with GTX 1080,	FINDeRS	315	Color fundus images	Accuracy = 95.71% Sensitivity = 76.92% Specificity = 100%
Recep E. Hacisoftaoglu et al. [123]	AlexNet, Google-Net, and ResNet-50-based transfer learning approach	MATLAB	EyePACS, MESSIDOR-1, IDRiD, and MESSIDOR-2, University of Auckland Diabetic	38,532	Color fundus images	Accuracy = 98.6% Sensitivity = 98.2% Specificity = 99.1%
ZUBAIR KHAN et al. [124]	VGG16, spatial pyramid pooling layer (SPP), and network-in-network (NiN)-based model	NVIDIA Tesla k40, Keras, and Tensor Flow	EyePACS	88,702	Color fundus images	Maximum accuracy = 85%, recall = 55.6% Specificity = 91% Precision = 67% F1-score = 59.6%

**Table 4:** Summary of techniques reported for the classification of lesions in DR images.

Study	Proposed Solution	Languages/Libraries Software/Tools for Simulation Environment and Implementation	Data Set	Number of Images Used	Image Modalities	Evaluation
Baidaa Al-Bander at al. [126]	End-to-end CNN model	Python and Theano libraries implemented on a NVIDIA GTX TITAN X 12 GB GPU card with 3072 CUDA with 4 GB Ram	MESSIDOR	1200	Colored fundus images	Accuracy = 88.8 % Sensitivity = 74.7% Specificity = 96.5 %
Daniel Shu Wei Ting et al. [127]	CNN-based model	Not mentioned	Custom-developed in Singapore National Diabetic Retinopathy Screening Program (SIDRP)	494,661	Not mentioned	AUC = 93.6% Sensitivity = 90.5% Specificity = 91.6%
Jordi de la Torre et al. [129]	Fully convolutional neural network (FCNN)	Not mention	EyePACS	88,650	Colored fundus images	Sensitivity = 91.1% Specificity = 90.8%
Abdüssamed Erciyas and Necaattin Barışçı [134]	Region-based fast CNN (RFCNN) and CNN-based method	Not mention	MESSIDOR and DIARETDB	11,711	Colored fundus images	Accuracy = 99.9% Sensitivity = 99.1%
Wejdan L. Alyoubi et al. [137]	CNN512 and YOLOv3-based model	Python and Keras and Tensor Flow on NVIDIA Tesla K20 GPU with 5 GB memory	DDR and Asia Pacific Tele-Ophthalmology Society (APTOS)	51,532	Colored fundus images	Accuracy = 89% sensitivity = 89% specificity = 97.3%
D. Jude Hemanth et al. [138]	Histogram equalization and contrast limited adaptive histogram equalization + CNN	MATLAB r2017a executed on Intel R9 Core i5-3230 M, 2.60 GHz CPU, 8 GB RAM	MESSIDOR	1200	Color fundus images	Accuracy = 97% Sensitivity (recall) = 94% Specificity = 98% Precision = 94% F-score = 94% GMean = 95%
Suvajit Dutta et al. [139]	Convolutional neural network (CNN), back propagation neural network (BPNN), deep neural network (DNN) and fuzzy c-means-based knowledge model	Not mentioned	Fundus images, Kaggle	2000	Colored fundus images	Maximum accuracy = 82.3%
P. Burlina et al. [140]	DCNN and linear support vector machine (LSVM)-based model	Not mentioned	NIH AREDS	5600	Colored fundus images	Accuracy = 95.0% Specificity = 95.6% Sensitivity = 93.4% Positive predictive value (PPV) = 89.6% Negative predictive value (NPV) = 97.3%
Ramzi Adriman et al. [142]	Local binary patterns (LBP)+ResNet-based system	NVIDIA^®^ GeForce GTX 1050Ti with memory 4 GB + PyTorch 1.2	APTOS 2019 Blindness	5592	Colored fundus images	Accuracy=96.36%
Xiangji Pan et al. [144]	Stochastic gradient descent (SGD) and DenseNet-based approach	Not mentioned	Custom-developed at Hospital of Zhejiang University School of Medicine from August 2016 to October 2018	4067	Fundus fluorescein angiography (FFA)	Maximum AUC = 96.53% Specificity = 99.5% Sensitivity = 80.3%
R. Arunkumar and P. Karthigaikumar [145]	Deep belief neural network (DBNN), generalized regression neural network (GRNN), and SVM-based framework	Not mentioned	ARIA	143	Colored fundus images	Accuracy = 96.73% Specificity = 97.89% Sensitivity = 79.32%
Charu Bhardwaj et al. [147]	Deep neural network and Inception-ResNet-v2-based framework	Not mentioned	MESSIDOR and IDRiD	Not mentioned	Colored fundus images	Accuracy = 93.33%
Thippa Reddy Gadekallu et al. [148]	Firefly-principal component analysis and deep neural network-based model	Python	Massidor	1151	Color fundus images	Accuracy = 96% Precision = 95% Recall = 95% Sensitivity = 90.4% Specificity = 94.3%
Yo-Ping Huang et al. [149]	Fuzzy analytical network and transformed fuzzy neural network-based method	Not mentioned	MESSIDOR	1151	Colored fundus images	Accuracy = 100%
A.B. Aujih et al. [150]	U-Net model	Intel Xeon, 16 cores, Nvidia GeForce GTX 1080ti, Ubuntu16.04	MESSIDOR and DRIVE	190,000	Colored fundus images	Accuracy = 97.72%
Emmy Bhatti and Prabhpreet Kaur [151]	Gaussian filter (GF) and multi-support vector machine (MSVM)-based method	MATLAB	DIARETDB0	348	Colored fundus images	Accuracy = 82% Specificity = 82% Sensitivity = 82.66%
Hongyang Jiang et al. [152]	Weighted class action maps (CAMs) + Ada boost-based system	Cloud server with Ubuntu system of 16.04 LTS amd64 (64 bit). Intel Xeon E5-2620 v3 processor of six 2.40 GHZ cores and 40 GB memory, a NVIDIA Tesla P40 of 24 GB memory and a local hard disk of 200 GB	Custom-developed at Beijing Tongren Eye Center	30,244	Colored fundus images	Accuracy = 94.6% Specificity = 90.85% Sensitivity = 85.57%
Xiaofei Wang et al. [153]	Deep multi-task DR grading (Deep MT-DR) model	Intel(R) Core(TM) i7-4770 CPU@3.40 GHz, 32 GB RAM and 4 Nvidia GeForce GTX 1080 Ti GPUs.	DDR and EyePACS	102,375	Colored fundus images	Accuracy = 88.7 Kappa=86.5
A. Rosline Mary and P. Kavitha [155]	Stochastic coordinate descent deep learning (SCDDL) architecture	2.8 GHz with Turbo Boost Up to 3.8 GHz, Intel Core i5-7700HQ, 8 GB DDR4 SDRAM, NVIDIA GeForce GTX 1050	APTOS, DDR, EPAC, DRIVE, and EOPTHAMA	Not Mentioned	Colored fundus images	Maximum accuracy = 97.83%
K. Shankar et al. [156]	DCNN and synergic network (SN)-based model	Not mentioned	MESSIDOR	1200	Colored fundus images	Accuracy = 99.28% Sensitivity = 98% Specificity = 99%
K. Shankar et al. [157]	Hyper-parameter-tuning Inception-V4 (HPTI-V4) and feed-forward artificial neural network (ANN)-based model	Python and Tensor Flow	MESSIDOR	1200	Colored fundus images	Accuracy = 99.49% Sensitivity = 98.83% Specificity = 99.68%
Teresa Araújo et al. [158]	Gaussian sampling approach and multiple instance learning-based DR|GRADUATE system	Intel Core i7-5960X, 32 Gb RAM, 2 × GTX1080 desktop with Python 3.5, Keras 2.2 and TensorFlow 1.8.	MESSIDOR-2, IDRID, DMR, SCREEN-DR, and Kaggle DR	103,066	Color fundus images	Maximum quadratic-weighted kappa = 84%
Juan Wang et al. [159]	Squeeze-and-excitation (SE) network and forward neural networks-based hierarchical framework	Not mentioned	Custom-developed at Shenzhen SiBright Co. Ltd. (Shenzhen, Guangdong, China)	89,917	Color fundus images	Maximum quadratic-weighted kappa = 95.37%
Hongyang Jiang et al. [160]	ResNet and gradient-weighted class activation mapping (Grad-CAM)-based multi-label model	Intel Xeon CPUs of 2.40 GHz cores, 100 GB memory and one NVIDIA Tesla P40 GPU of 24 GB memory	MESSIDOR and custom-developed at Beijing Tongren Eye Center	3228	Color fundus images	Sensitive = 93.9% Specificity = 94.4% Accuracy = 94.2% AUC = 98.9%
Paisan Raumviboonsuk et al. [164]	Convolutional neural network with Inception-V4	Tensor Flow	Custom-developed at Bangkok Metropolitan Administration Public Health Center	25,326	Colored fundus images	Area under the curve (AUC) = 98.7% Sensitivity = 97% Specificity = 96%

**Table 5:** Summary of the techniques reported for the classification of lesions in DR images.

Study	Proposed Solution	Languages/Libraries Software/Tools for Simulation Environment and Implementation	Data Set	Number of Images Used	Image Modalities	Evaluation
Daniel Shu Wei Ting et al. [127]	Convolutional neural network-based learning system	Not mentioned	Custom-developed by Singapore National Diabetic Retinopathy Screening Program (SIDRP)	494,661	Color fundus Images	AUC = 93.6% Sensitivity = 90.5% Specificity = 91.6%
Pedro Romero-Aroca et al. [167]	CNN-based Algorithm	Not mentioned	EyePACS and MESSIDOR-2	90,450	Color fundus Images	Cohen’s weighted kappa (CWK) index = 88.6% Sensitivity = 96.7% Specificity = 97.6% Positive predictive value (PPV) = 83.6% Negative predictive value (NPV) = 99.6%

## Data Availability

Not applicable.
